# Host Defense against Viral Infection Involves Interferon Mediated Down-Regulation of Sterol Biosynthesis

**DOI:** 10.1371/journal.pbio.1000598

**Published:** 2011-03-08

**Authors:** Mathieu Blanc, Wei Yuan Hsieh, Kevin A. Robertson, Steven Watterson, Guanghou Shui, Paul Lacaze, Mizanur Khondoker, Paul Dickinson, Garwin Sing, Sara Rodríguez-Martín, Peter Phelan, Thorsten Forster, Birgit Strobl, Matthias Müller, Rudolph Riemersma, Timothy Osborne, Markus R. Wenk, Ana Angulo, Peter Ghazal

**Affiliations:** 1 Division of Pathway Medicine and Centre for Infectious Diseases, University of Edinburgh, Edinburgh, United Kingdom; 2 Centre for Systems Biology at Edinburgh, The King's Buildings, Edinburgh, United Kingdom; 3 Department of Biochemistry and Department of Biological Sciences, National University of Singapore, Singapore; 4 Institut d'Investigacions Biomediques August Pi i Sunyer, Barcelona, Spain; 5 Metabolic Signaling Diseases Program, Sanford-Burnham Medical Research Institute, Orlando, Florida, United States of America; 6 Institute of Animal Breeding and Genetics, Veterinary University of Vienna, Vienna, Austria; 7 Centre for Cardiovascular Disease, University of Edinburgh, Edinburgh, United Kingdom; Washington University School of Medicine, United States of America

## Abstract

Little is known about the protective role of inflammatory processes in modulating lipid metabolism in infection. Here we report an intimate link between the innate immune response to infection and regulation of the sterol metabolic network characterized by down-regulation of sterol biosynthesis by an interferon regulatory loop mechanism. In time-series experiments profiling genome-wide lipid-associated gene expression of macrophages, we show a selective and coordinated negative regulation of the complete sterol pathway upon viral infection or cytokine treatment with IFNγ or β but not TNF, IL1β, or IL6. Quantitative analysis at the protein level of selected sterol metabolic enzymes upon infection shows a similar level of suppression. Experimental testing of sterol metabolite levels using lipidomic-based measurements shows a reduction in metabolic output. On the basis of pharmacologic and RNAi inhibition of the sterol pathway we show augmented protection against viral infection, and in combination with metabolite rescue experiments, we identify the requirement of the mevalonate-isoprenoid branch of the sterol metabolic network in the protective response upon statin or IFNβ treatment. Conditioned media experiments from infected cells support an involvement of secreted type 1 interferon(s) to be sufficient for reducing the sterol pathway upon infection. Moreover, we show that infection of primary macrophages containing a genetic knockout of the major type I interferon, IFNβ, leads to only a partial suppression of the sterol pathway, while genetic knockout of the receptor for all type I interferon family members, *ifnar1*, or associated signaling component, *tyk2*, completely abolishes the reduction of the sterol biosynthetic activity upon infection. Levels of the proteolytically cleaved nuclear forms of SREBP2, a key transcriptional regulator of sterol biosynthesis, are reduced upon infection and IFNβ treatment at both the protein and de novo transcription level. The reduction in *srebf2* gene transcription upon infection and IFN treatment is also found to be strictly dependent on *ifnar1*. Altogether these results show that type 1 IFN signaling is both necessary and sufficient for reducing the sterol metabolic network activity upon infection, thereby linking the regulation of the sterol pathway with interferon anti-viral defense responses. These findings bring a new link between sterol metabolism and interferon antiviral response and support the idea of using host metabolic modifiers of innate immunity as a potential antiviral strategy.

## Abstract 2

Upon infection, our immune cells produce a small protein called interferon, which in turn signals a protective response through a series of biochemical reactions that involves lowering the cells' ability to make cholesterol by targeting a gene essential for controlling the pathway for cholesterol metabolism.

## Author Summary

Currently, little is known about the crosstalk between the body's immune and metabolic systems that occurs after viral infection. This work uncovers a previously unappreciated physiological role for the cholesterol-metabolic pathway in protecting against infection that involves a molecular link with the protein interferon, which is made by immune cells and known to “interfere” with viral replication. We used a clinically relevant model based on mouse cytomegalovirus (CMV) infection of bone-marrow-derived cells. Upon infection these cells produce high levels of interferon as part of the innate-immune response, which we show in turn signals through the interferon receptor resulting in lowering enzyme levels on the cholesterol pathway. We observed this effect with a range of other viruses, and in each case it leads to a notable drop in the metabolites involved in the cholesterol pathway. We found that the control mechanism involves regulation by interferon of an essential transcription factor, named SREBP-2, which coordinates the gene activity of the cholesterol pathway. This mechanism may explain clinical observations of reduced cholesterol levels in patients receiving interferon treatment. Our initial investigation into how lowered cholesterol might protect against viral infection reveals that the protection is not due to a requirement of the virus for cholesterol itself but instead involves a particular side-branch of the pathway that chemically links lipids to proteins. Drugs such as statins and small interfering RNAs that block this part of the pathway are also shown to protect against CMV infection of cells in culture and in mice. This provides the first example of targeting a host metabolic pathway in order to protect against an acute infection.

## Introduction

Sterols and fatty acids are common intermediary metabolites that play key roles in many biological pathways involved in inflammatory diseases such as atherosclerosis and chronic heart disease [1]–[4]. Significantly, mounting evidence shows a connection between innate immune signaling processes and the regulation of sterol and fatty acid metabolism [5]–[8]. Specifically, cholesterol and its metabolites have been shown to alter inflammatory mediator behavior [9]–[11], and conversely, innate immune signaling has been shown to modulate the dynamics of cholesterol transport, storage, and excretion [12]–[15]. Recent studies have also begun to show that the perturbation of lipid metabolism in a range of virally infected cells is a hallmark of cellular changes associated with infection. For instance, studies analyzing the consequences of human cytomegalovirus (CMV) infection have shown that increases in the flux of the fatty acid biosynthesis pathway are essential for optimal viral growth in fibroblasts [16]. Further, Hepatitis C virus (HCV) has been shown to co-opt the prenylation pathway to promote the efficient replication of its genome [17]–[19]. More generally, a number of other viruses, notably Measles, HIV, West Nile virus, and Dengue virus, also have the ability to change cholesterol pathway gene expression in a variety of cellular systems [20]–[24]. Whether the effects of virus infection on the cholesterol pathway are directly mediated by the pathogen or indirectly host-mediated mechanisms is not known. From a therapeutic perspective, studies have also shown that the pharmacological disruption of the cholesterol metabolism by statins and other metabolic modifiers can result in the inhibition of viral replication [25]–[32].

It is well documented that the cross-talk between immune programs of macrophage activation and lipid homeostasis plays a central part in chronic inflammatory diseases [33],[34]. In particular an anti-atherosclerosis transcriptional axis of PPARγ regulating a pathway of cholesterol efflux by inducing ABCA1 expression and cholesterol removal from macrophages, via a transcriptional cascade mediated by activated LXRα, has been reported [35]. Significantly, cellular metabolic, signaling, and regulatory pathways also play a critical “collaborative” role in modulating immune responses to infection [36]. In this context, Toll-like pathogen recognition receptors, crucial to the initiation of innate immune signaling, have recently been shown to regulate the expression of key lipid-associated genes following bacterial infection. This occurs due to microbial ligand activation of the IRF3 pathway, which blocks the induction of LXR target genes such as ABCA1 and inhibits cholesterol efflux from macrophages in an interferon independent manner [5]. In this context, LXRα−/− mice are more susceptible to bacterial infection [37], further emphasizing the importance of this pathway in the innate immune response. From a viral perspective, an interferon-inducible protein “viperin” is known to inhibit influenza A virus and HCV by disrupting the formation of cholesterol-enriched lipid rafts, which act as attachment sites for viral production [38],[39]. Significantly, despite increasing numbers of studies in this area, the question remains as to whether the immune regulation of lipid pathways can also serve a role as part of a protective anti-viral response. Indeed, in the context of host protection pathways, it is not known whether a central immune regulatory mechanism involving interferon response is directly or indirectly required in modulating lipid metabolism in infection.

We are interested in elucidating the relationship between transcriptional networks and immune regulatory pathways and host-cell dependency mechanisms of pathogens, especially viruses, as identifying host dependency mechanisms at the pathway level provides a new molecular systems-level approach for understanding viral pathogenesis, which can be harnessed as an anti-infective strategy [40]–[42]. For many years, studies of virus-host interactions, in particular for large DNA viruses, have proven invaluable in the characterization of host cell molecular pathways and their connectivity to the inflammatory response. Murine cytomegalovirus (mCMV), which has a large double-stranded DNA genome, represents one of the few model organisms studied in its natural host and has both biological and clinical relevance to human CMV disease [43]. In this study, we have sought to apply a systems-level approach, bringing together functional genomics, lipidomics, and biochemical experimentation, to understand the interplay between sterol pathway down-regulation and the innate immune response to mCMV infection. Our investigations reveal a previously undisclosed dependency role for down-regulation of the sterol metabolic network, which is integral to the protective immune response requiring a type 1 interferon receptor regulatory loop mechanism.

## Results

### Sterol Biosynthesis Pathway–Associated Genes Are Co-ordinately Down-Regulated by IFNγ Treatment and mCMV Infection in Primary Bone-Marrow–Derived Macrophages

**Figure 1:**
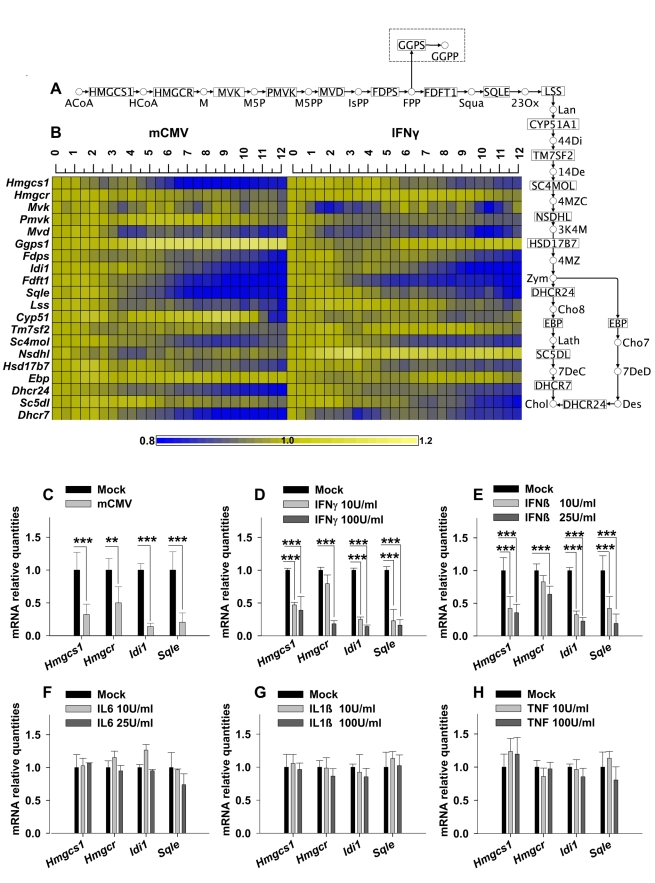
Regulation of the cholesterol pathway upon mCMV infection. (A) The Sterol biosynthesis pathway shown in KEGG notation with abbreviated metabolites (abbreviations listed in Text S1). The geranylgeranylation pathway responsible for GGPP synthesis is shown in the dashed box. (B) Heat map of the cholesterol biosynthesis temporal genes' expression during the first 12 h of mCMV infection (left panel) or IFNγ treatment (right panel). Each time point corresponds to one independent biological sample, and columns indicate time in hours. Fold changes of expression levels are represented on a Log2 scale compared to mock-treated cells, ranging from a 0.8× lower expression (dark blue) to a 1.2× higher expression (bright yellow). (C–H) Expression analysis measured by qRT-PCR of *Hmgcs1*, *Hmgcr*, *Idi1*, and *Sqle* genes in BMDM infected with mCMV(24 hpi) (C) or treated for 24 h with IFNγ (10 and 100 U/ml) (D), IFNβ (10 and 25 U/ml) (E), IL6 (10 and 25 U/ml) (F), IL1β (10 and 100 U/ml) (G), or TNF (10 and 100 U/ml) (H). Graphs show levels of mRNA expression of the respective genes either infected or cytokines-treated relative to mock samples. Bars represent the means ± SD of five independent experiments with biological triplicates for each experiment. *p<0.05, **p<0.01, ***p<0.001, determined with an unpaired Student's *t* test.

As a first step, an integrative approach combining bioinformatics tools and a time-series analysis of gene expression changes was applied to mCMV-infected or interferon (IFN) γ-activated primary bone-marrow-derived macrophages (BMDM). These primary BMDM cultures represent a physiologically relevant cell system for the combined analysis of infection, inflammation, and lipogenesis [44]–[46]. In the following experiments, infected or IFNγ-treated BMDM RNA was harvested every 30 min up to 12 h post-challenge for microarray gene expression profiling. In this study, analysis of expression data was exclusively restricted to lipogenic-associated genes. For this purpose, a combination of literature and data-mining identified over one thousand genes with published direct or indirect functions relating to cellular lipid metabolism, regulation, and synthesis (Text S1). When this resource was used to interrogate a subset of our time-series data which passed a stringent filtering threshold (*p*<10^−6^), 89% of lipogenic-associated genes were detected, of which 12% were significantly regulated (113/958) upon IFNγ treatment and 23% were significantly altered in their expression (195/958) after mCMV infection. This represented a significant and highly selective lipogenic response (Figure S1) with altered genes showing a high degree of overlap between infection and IFNγ activation (Table S1). Notably, clear differences in the specific class of lipogenic genes in up- and down-regulated groups were observed. Of the IFNγ down-regulated transcripts, a significant proportion (14/35, 40%) were related to the sterol pathway, while fatty acid pathways were pre-eminent (6/35, 17%) in the up-regulated gene group (Figure S1C). A statistical evaluation investigating pathway over-representation indicated a highly pathway-specific response including previously known pathways for inositol (Table S2E–F) [47] perturbed by mCMV infection. Significantly, however, the most pronounced pathway changes in the down-regulated genes common to both stimuli were associated with sterol lipid metabolism (Table S2E and Figure 1A), which exhibited a gradual, temporal decline in expression from 6 h post-infection (hpi) onwards (Figure 1B). Additional microarray experiments to further explore this observation revealed a further reduction in sterol pathway gene expression observed at 24 hpi (unpublished data). It is worth noting, however, that the observed level of reduction in expression for any particular transcript was relatively modest (ranging from 1.3- to 5-fold for infection and 1.3- to 3-fold for IFNγ treatment over a 24 h time frame).

**Figure 2:**
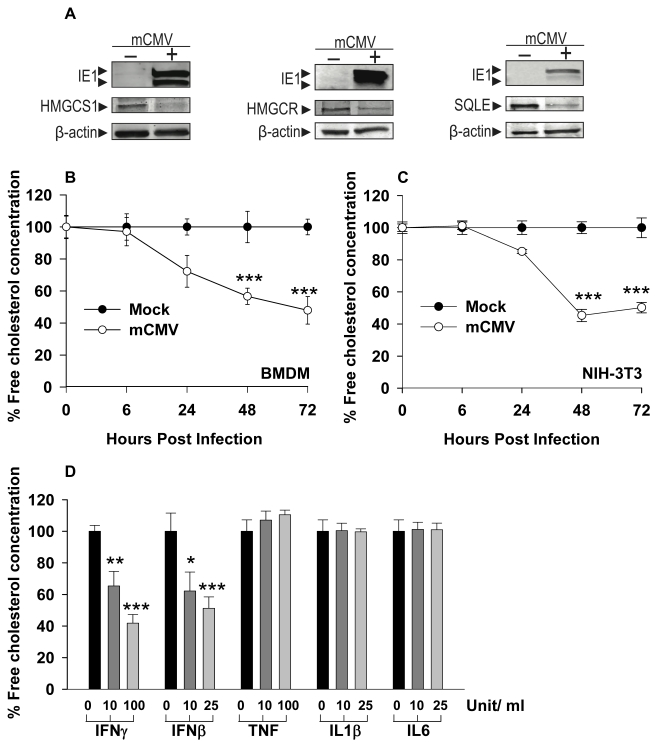
Effect of a coordinated reduction in multiple enzymes on sterol biosynthesis. (A) Comparison by Western blot analysis of HMGCS1, HMGCR, and SQLE protein levels in mCMV infected (24 hpi) or mock-treated BMDM. Infection was measured by detection of the IE1 mCMV antigen. Intensity values relative to β-actin calculated by densitometry show a decrease of the total amount of protein in the mCMV-infected BMDM compared to the mock-treated samples of 64% for HMGCS1, 50% for HMGCR, and 85% for SQLE. Graphs are representative of two independent experiments with biological duplicates and triplicates, respectively. (B–C) Free cholesterol concentration was determined experimentally by enzymatic assay (Materials and Methods) at 0, 6, 24, 48, and 72 hpi in BMDM (B) and NIH/3T3 cells (C). Cholesterol content is presented as the percentage of free intracellular cholesterol concentration from infected cells compared to mock treatment. Graphs represent the means ± SD of three independent experiments with biological quadruplicates for each experiment. (D) Free cholesterol concentration in BMDM cultures treated with varying concentrations of IFNγ, IFNβ, TNF, IL1β, or IL6. The cholesterol concentration was measured as mentioned above after 48 h post-cytokine treatment. Bars represent means ± SD of two independent experiments with biological quadruplicates for each experiment. *p<0.05, **p<0.01, ***p<0.001, determined with an unpaired Student's *t* test.

To independently validate the microarray data described above, Q-RT-PCR analyses of five independent experiments were performed for both infection and IFNγ treatment. In agreement, we find that Q-RT-PCR analysis of selected members of the pathway—*Hmgcs1*, *Hmgcr*, *IdI1*, and *Sqle*—shows a statistically significant but quantitatively modest reduction in expression (Figure 1C and 1D). Notably, a similar quantitative decrease is also exhibited at the protein level for HMGCS1, HMGCR, and SQLE (Figure 2A).

### Down-Regulation of the Sterol Biosynthesis Pathway Is Specific to IFNγ and IFNβ Treatment

Since the alterations in expression of the cholesterol-related genes were consistent but of relatively small magnitude, we considered whether these perturbations represented either non-specific “noise” generated during the pro-inflammatory stimulation of a macrophage or a more specific response to a particular challenge. To test whether alternative pro-inflammatory mediators could also lead to the modulation of the sterol pathway genes, macrophage cultures were treated with a range of doses of the following inflammatory cytokines: IL1β, TNF, IL6, and IFNβ. *Hmgcs1*, *Hmgcr*, *Idi1*, and *Sqle* gene expression changes were then analyzed by Q-RT-PCR (Figure 1E–H). Of the cytokines tested, only IFNβ elicited the down-regulation of sterol pathway gene expression in primary macrophage cultures (Figure 1E). In summary, these data indicate a highly specific response of macrophages through a coordinated negative regulation of multiple sterol pathway members upon viral infection or treatment with IFNγ or β but not IL1β, TNF, or IL6. Once again, these effects are quantitatively “modest” but statistically significant.

### Experimental Testing of Bioinformatic Predictions: Infection Results in a Decrease of Sterol Metabolites in Primary Macrophages and Fibroblasts

We next sought to explore how multiple small reductions in enzyme levels impact upon the biosynthetic activity of the pathway by measuring the steady-state metabolic output of the pathway. For these experiments, free intra-cellular cholesterol level, as a metabolic end product of the sterol pathway, was determined using an enzymatic method on infected macrophages (Figure 2B). We observe a significant decrease in cholesterol metabolite levels 24 hpi. Similar results were also observed with infection of NIH/3T3 cells (Figure 2C), indicating that the effect is not macrophage specific.

It is possible that the experimentally observed drop in sterol lipid levels could be due to a non-specific and generalized response to infection, although from the microarray analysis of the lipidomic associated genes we clearly observe highly specific lipogenic responses rather than a broad response to infection (Figures S1 and S2). To further determine whether the down-regulation of sterol biosynthesis is specific between mCMV infection and select lipogenesis pathways, total cell extracts were analyzed by electrospray ionization as well as atmosphere chemical ionization mass spectrometry (see Materials and Methods). These lipidomic approaches allow quantification of the major membrane lipid classes (such as glycerophospholipids and sterols) as well as individual molecular lipid species at high sensitivity. Overall, we find no coordinated or substantial differences in the overall levels of major glycerophospholipids (phosphatidylcholine, phosphatidylserine, and phosphatidylethanolamine) during infection with CMV, although a small number of the individual species in the phosphatidylcholine and phosphatidylserine group are affected (Figure S3A–C). In marked contrast, levels of free cholesterol, as well as its immediate precursor, zymosterol, 14-demethyl-lanosterol, and 7-dehydrocholesterol, were strongly reduced at 24 hpi (2–3-fold) and 48 hpi (4–6-fold) (Figure S2A–D). These results further support a specific alteration of sterol biosynthesis upon infection. Furthermore, the reduced free cholesterol levels are also developed in a dose-dependent manner by treatment with IFNβ and γ but not IL1β, IL6, or TNF (Figure 2D). Altogether, we conclude that the effect of the coordinated down-regulation is to reduce metabolic output of the sterol pathway.

### Pharmacologic Inhibition and siRNA Knock-Down of the Sterol Biosynthesis Pathway Has an Antiviral Effect

**Figure 3:**
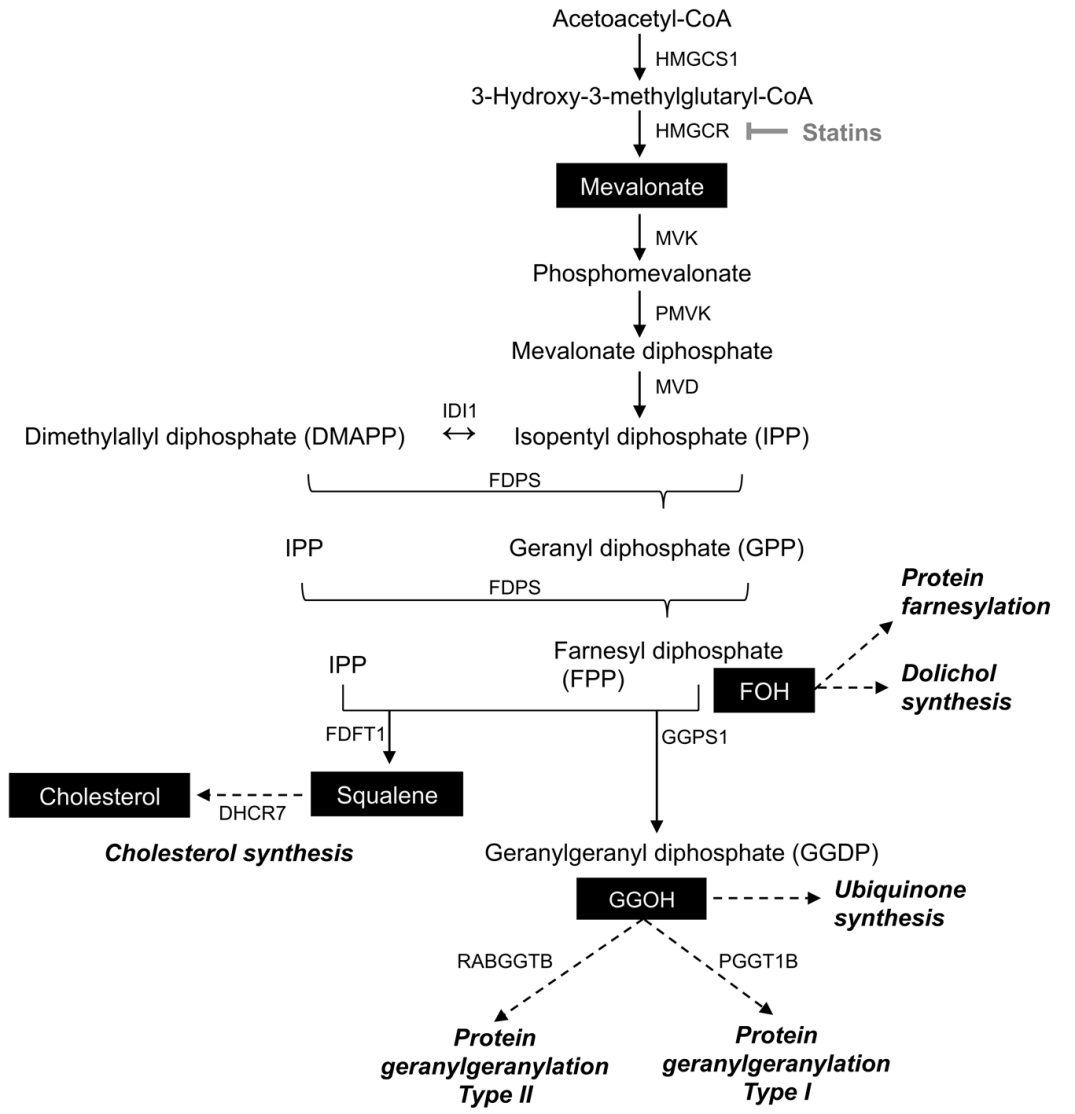
Schematic of the mevalonate-isoprenylation branch point of the sterol biosynthesis pathway. Metabolites (shown in inverse print) and inhibitor (Simvastatin) (shown in grey) used to dissect the pathway are indicated: Simvastatin inhibits HMGCR and prevents the synthesis of mevalonate and downstream lipids.

**Figure 4:**
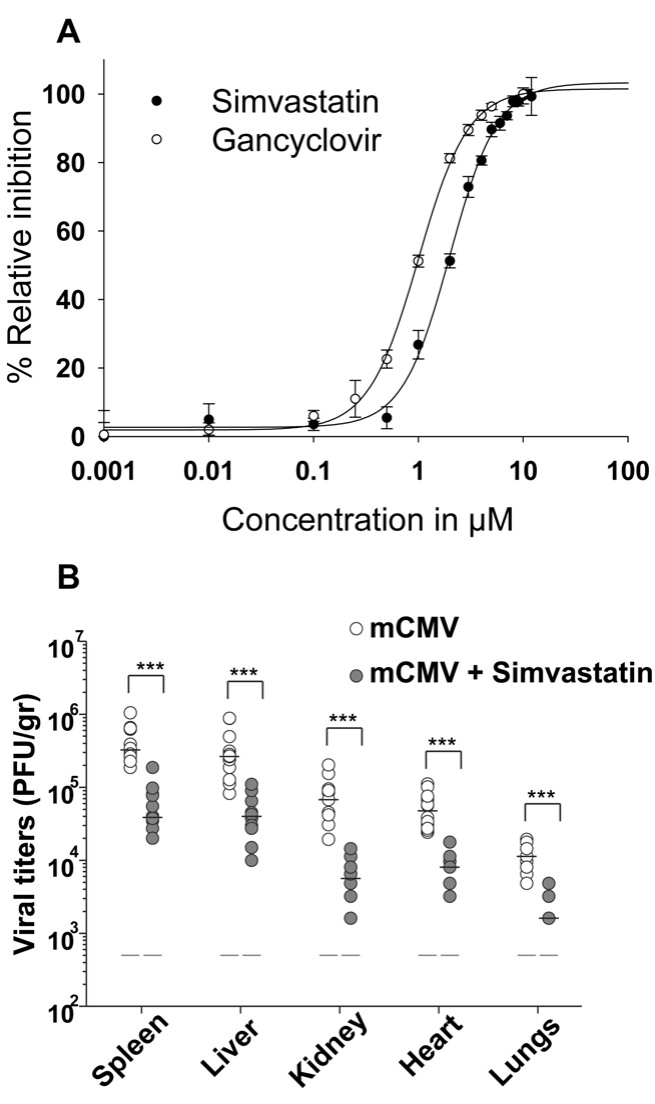
Effect of statins on mCMV growth in vitro and in vivo. (A) NIH/3T3 cells were infected with mCMV-GFP (MOI of 0.2) and subsequently treated with varying concentrations of Simvastatin or Gancyclovir immediately after infection. GFP expression was measured to monitor the level of infection (Materials and Methods). Graph represents the percentage of viral inhibition as a function of drug treatment. Data points represent mean ± SD of two independent experiments with six biological replicates for each experiment. (B) Mice were fed with simvastatin (50 mg/kg/mice) daily for 5 d by gavages and at day 1 post-treatment, were challenged with 2×10^6^ PFU of mCMV by intraperitoneal injection, and sacrificed; at 4 dpi, viral titers in different organs were measured by plaque assay and are expressed per gram of tissue. Data points represent mean ± SD of two independent experiments with five mice per group for each experiment. *p<0.05, **p<0.01, ***p<0.001, determined with a Mann-Whitney U test.

To assess whether the sterol biosynthesis pathway plays a pro- or anti-viral role in regulating mCMV replication, we exploited the pharmacologic compound “simvastatin,” a potent and selective inhibitor of HMGCR [48]. Inhibition of HMGCR is known to result in a reduction of the metabolic intermediate mevalonate (Figure 3) and an accompanying drop in cholesterol synthesis by the cell [49]. The treatment of cells with simvastatin resulted in a dose-dependent inhibition of mCMV plaque formation (unpublished data) and in live cell replication assays (Figure 4A) with an IC50 of 2 µM that is comparable to the “gold standard” anti-viral Gancyclovir (Figure 4A) in the murine model system. Notably, the observed inhibitory effect of simvastatin occurred below a level at which non-specific toxic effects to cells were observed (15 µM) (Figure S7). These experiments pointed to a potential protective anti-viral role via a targeted disruption of the sterol pathway and raised the question of whether pharmacologic treatment in vivo also develops an inhibitory effect. To investigate whether simvastatin could play an anti-infective role in vivo, mice were administered with an established pre-clinical pharmacologic dose of simvastatin or vehicle alone and infected by intra-peritoneal inoculation with mCMV. Viral titres were then determined in a variety of organs at day 4 post-inoculation. Markedly, viral titres are reduced by over one order of magnitude in multiple organs following treatment with simvastatin (Figure 4B).

**Figure 5:**
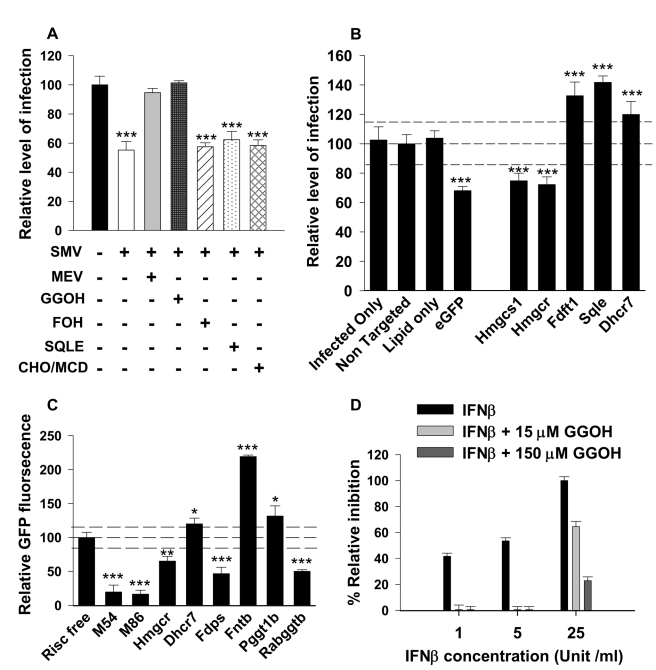
Metabolic investigation of the sterol pathway in infection. (A) NIH/3T3 cells were infected with mCMV-GFP (MOI of 0.2) and subsequently treated with Simvastatin (SMV) (2.5 µM) and mevalonate (MEV) (300 µM) or geranylgeraniol (GGOH) (15 µM) or farnesol (FOH) (15 µM) or squalene (SQE) (15 µM) or of water soluble cholesterol (complexes of cholesterol with methyl-β-cyclodextrin, CHO/MCD) (5 µg/ml) for 72 h. The level of infection was determined by measuring GFP fluorescence at 76 hpi (Material and Methods). Graph represents the relative level of infection compared to the untreated cells, and bars represent mean values ± SD of three independent experiments with five biological replicates for each experiment. (B) NIH/3T3 cells were transfected for 48 h with either non-targeted, eGFP, Hmgcs1, Hmgcr, Sqle, Fdft1, or Dhcr7 On-target plus siRNA smart pool and then infected with mCMV-GFP (MOI of 0.2). (C) NIH/3T3 cells were transfected for 48 h with either Risc Free, M54, and M86 (knocking down mCMV viral genes), Hmgcr, Dhcr7, Fdps, Fntb, Pggt1b, or Rabggtb On-target plus siRNA smart pool, and then infected with mCMV-GFP (MOI of 0.2). The level of non-targeted siRNA (B) and Risc Free (C) treated cells was used as a baseline estimate for the cutoff point (two standard deviations and a *p* value <0.001 (determined with an unpaired Student's *t* test) as significant). Bars represent means ± SD of two independent experiments with three biological replicates for each experiment. (D) NIH/3T3 cells were incubated with various doses of IFNβ for 18 h in the presence or absence of 15 and 150 µM GGOH. The graph represents the inhibition of viral replication (in percentages) as a function of drug concentration. Bars represent mean ± SD of biological triplicates for each experiment. *p<0.05, **p<0.01, ***p<0.001, determined with an unpaired Student's *t* test.

To determine the extent of the overlap between the sterol biosynthesis pathway and anti-viral activity, we employed a series of metabolite rescue and interference RNA knock-down experiments. In these experiments we observed that simvastatin anti-viral activity could be completely reversed by the addition of mevalonate to cells in culture (Figure 5A). This showed that the anti-viral mechanism was due to an inhibition of HMGCoA reductase. While this result supports the requirement of the mevalonate arm of the sterol pathway, it does not necessarily implicate cholesterol as being responsible for the anti-viral activity. Notably, feeding macrophages with a cell permeable form of cholesterol or squalene failed to reverse the inhibitory activity (Figures 3 and 5A), indicating that the anti-viral effect is unlikely to be cholesterol mediated and thus unrelated to any regulatory sterols or to the structural requirements of virus replication associated with lipid droplets. The addition of cell permeable farnesol also did not rescue the inhibitory activity of simvastatin, while conversely the addition of geranylgeraniol fully rescued the anti-viral activity (Figures 3 and 5A). These experiments show the specificity of the metabolic requirement for anti-viral activity and highlight a possible role for the mevalonate-isoprenoid arm of the sterol pathway in protection against mCMV infection (Figure 3).

While the rescue of statin inhibition of viral growth by mevalonate and geranylgeraniol strongly indicates the involvement of the proximal arm of the sterol pathway, it is still conceivable that other mechanisms of action unrelated to the capacity to inhibit biosynthesis may be responsible for the effect on virus replication. For this reason and to additionally test the specific requirement of members of the sterol biosynthesis pathway for viral growth control, siRNA knock-down experiments were performed. For these experiments, *Hmgcs1* and *Hmgcr* were first targeted in the pathway using low concentrations of siRNA to avoid non-specific interferon responses, including a series of non-targeting siRNA for non-targeting effects. Knock-down of these genes (Figure S5) resulted in a specific and significant decrease in the optimal rate and end-point yield of viral replication (Figures 5B). To further dissect the specific role of pathway members in mediating the anti-viral response, additional siRNA inhibition studies were conducted involving targeted genes distal to the mevalonate-prenylation branch of the sterol biosynthesis pathway. In these experiments, Fdft1, Sqle, and Dhcr7 were targeted. Figure 5B clearly shows that targeting these members of the pathway fails to inhibit and even positively influences viral growth, a result that is consistent with the above described metabolite rescue experiments. To further investigate and to independently assess the specificity of the prenylation branch of the pathway, additional siRNA knock-down experiments were performed targeting farnesyl diphosphate synthase (Fdps), an enzyme essential for isoprenoid biosynthesis, and all three prenyltransferases (these are farnesyltransferase, geranylgeranyltransferase type I, and Rab geranylgeranyltransferase type II enzymes). In these experiments knock-down of Hmgcr and Dhcr7 and viral ORFs (M54 and M86) are used as controls and developed the expected knock-down profile (Figure 5C). Notably, significant inhibition of viral replication is observed for knock-down of Fdps. In the case of the downstream prenyltransferases, reduced viral replication is observed with siRNA targeting Rabggtb specific for geranylgeranyltransferase type II enzyme, but not Pggt1b or Fntb specific for gernylgeranyltransferase type I and farsenyltransferase, respectively (Figure 5C). These experiments indicate specificity of targeting the isoprenoid pathway but will require further functional validation work.

Overall, these findings show that inhibition of viral growth is not due to cholesterol deprivation, but rather a part of the pathway involving a proximal mevalonate-prenylation step. This raises the notion of whether depletion of geranylgeraniol may be one potential mode for interferon to inhibit viral replication. In this scenario we might expect that feeding cells with geranylgeraniol upon interferon treatment would counter, in part, the anti-viral effect. To determine the effect of interferon on viral replication, in the absence and presence of geranylgeraniol, we performed a metabolite rescue experiment in the presence of increasing units of IFNβ. Figure 5D shows that the anti-viral effect of 1 and 5 U/ml of IFNβ is dramatically reduced in the presence of geranylgeraniol (at both 15 and 150 µM), while at a more potent level of IFNβ (at 25 U/ml) approximately 70% and 25% of the anti-viral activity remains with 15 and150 µM GGOH, respectively. Taken together, these results support a role of the mevalonate-isoprenoid arm of the sterol pathway for optimal mCMV replication and highlight the potential role for down-regulating this pathway in protecting the host from viral infection. These findings also suggest that sterol biosynthesis regulation acts as a marker for antiviral activity.

### An Interferon Regulatory Loop Is Responsible for the Transcriptional Down-Regulation of the Sterol Biosynthesis Pathway in Response to Infection

**Figure 6:**
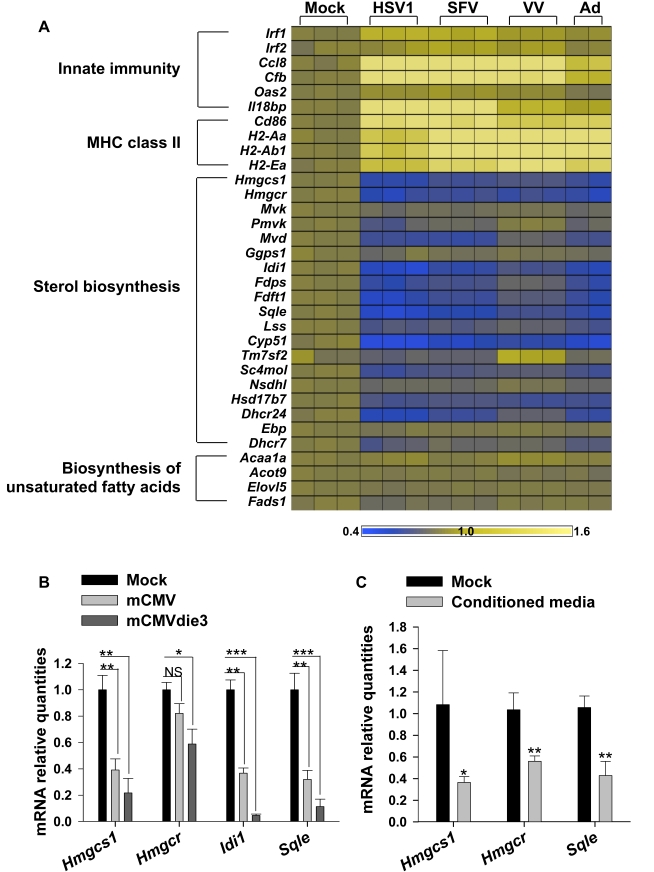
Alteration of gene expression upon HSV1, SFV, VV, adenovirus, and non-infectious mCMV in primary macrophages. (A) Heat map of expression levels of a set of genes after 24 h mock treatment, infection with Herpes simplex virus 1 (HSV1), Semliki forest virus (SFV), Vaccinia virus (VV), or Adenovirus (Ad) in BMDM (Text S1). Genes represent the innate immunity activation, the MHC class II antigen presentation, and the cholesterol and unsaturated fatty acids biosynthesis. Each square represents a single biological replicate. Fold changes of expression levels are represented on a Log2 scale compared to mock-treated cells, ranging from a 0.4× lower expression (dark blue) to a 1.6× higher expression (bright yellow). (B) Expression analysis measured by qRT-PCR of *Hmgcs1*, *Hmgcr*, *Idi1*, and *Sqle* genes in BMDM after 24 h mock treatment, mCMV, or mCMVdie3 infection, respectively. Graphs show the level of expression of the indicated genes relative to mock-treated samples and bars represent mean ± SD of two independent experiments with triplicate biological measurements for each experiment. (C) BMDM were infected with mCMV or mock treated, and supernatant was collected after 8 h and directly added to fresh BMDM. After 24 h, RNA from these cultures was collected and *Hmgcs1*, *Hmgcr*, and *Sqle* expression were measured by qRT-PCR. To test for the presence of any detectable virus, an aliquot of the supernatant was used to perform a standard plaque assay (no infectious virus detected, unpublished data). Graphs show the level of expression of the indicated genes relative to mock-treated samples and bars represent means ± SD of three independent experiments with triplicate biological measurements for each experiment. *p<0.05, **p<0.01, ***p<0.001, determined with an unpaired Student's *t* test.

We next sought to investigate whether specific viral or cellular modes of action might be responsible for the reduction in sterol biosynthesis upon infection. First, it is possible that the effects monitored in our experimental system are specific to mCMV. To test whether the down-regulation of sterol pathway gene expression is a more general effect rather than specific to mCMV, primary macrophages (BMDM) were infected with a number of different viruses and harvested for gene expression analysis. Figure 6A shows the expression profile of the sterol pathway and other pathways for infection (innate immune activation pathways) by an enveloped DNA virus, herpes simplex (HSV1); an RNA virus, semliki forest virus (SFV); cytoplasmic DNA virus, vaccinia virus (VV); and non-enveloped nuclear DNA virus, adenovirus. All the viruses tested show a specific and coordinate decrease in gene expression for members of the sterol biosynthesis pathway.

In the case of mCMV, it is worth noting that the reduction in gene expression occurs approximately 6 hpi (e.g., see Figure 1). Consequently, it is possible that a viral early or late gene product may be required for the effect. To test this possibility, we used a replication and early/late gene defective mCMV virus (mCMVdie3 in [50]). The mCMVdie3 strain is capable of infecting cells at levels equivalent to wild-type virus but is incapable of expressing its genome downstream of a rather restricted immediate-early phase. The results of these experiments are shown in Figure 6B, in which mCMVdie3 potently develops an equivalent level of down-regulation of sterol genes as the parental wild-type and revertant viruses, respectively.

It is well established for many viruses, including mCMV, that infection leads to the induced expression of type 1 interferon and pro-inflammatory cytokines. Two signaling cascades—a virus-induced interferon-producing signal and an interferon receptor-mediated secondary signal—regulate the interferon system. The first is initiated by the detection of viral components by host recognition receptors (PRRs) and leads to the activation of transcription factors—NFkB, ATF2/c-Jun, IRF3, and IRF7—that activate IFNα and β genes. The expressed interferons then transmit a secondary autocrine or paracrine signal through interactions with type I receptors that activate the JAK-STAT pathway. In this context, the above studies with the combined observation that interferon treatment and the cell response to infection are equally capable of causing a down-regulation of the sterol metabolic pathway raise the question of whether infection-mediated regulation might result from an interferon regulated loop. In support of this notion we find that low multiplicities of infection still exhibit a significantly reduced level of free cholesterol (Figure S6) and that conditioned media from infected macrophages 8 hpi (prior to release of any new viral particles) are sufficient to down-regulate the sterol biosynthesis pathway genes in uninfected control cultures (Figure 6C).

**Figure 7:**
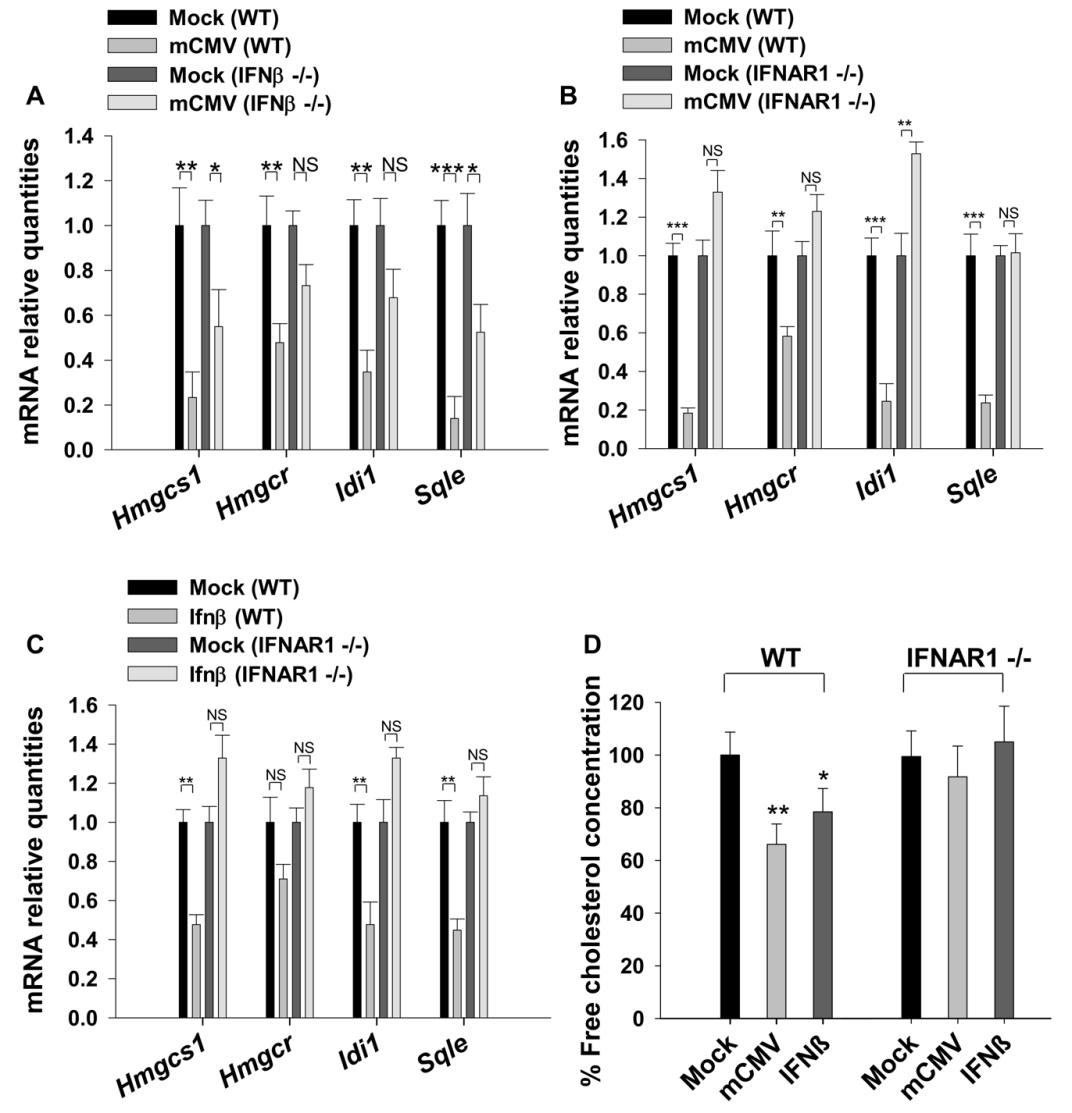
Contribution of type I interferon response in the regulation of sterol biosynthesis genes upon infection. (A–C) Wild type BMDM or BMDM from IFNβ−/− knockout mice or from IFNAR1−/− knockout mice were mock treated, infected with mCMV, or treated with IFNβ (10 U/ml) for 24 h. RNA was collected and the gene expression of *Hmgcs1*, *Hmgcr*, *Idi1*, and *Sqle* was measured by qRT-PCR. Graphs show the level of expression of the indicated genes relative to mock-treated samples. Bars represent the mean ± SD of biological quadruplicates. (D) Wild type BMDM or BMDM from IFNAR1−/− knockout mice were infected with mCMV or treated with IFNβ (10 U/ml). After 48 h, free cholesterol concentration was measured by enzymatic assay (Materials and Methods). Bars represent the mean ± SD of biological quadruplicates. *p<0.05, **p<0.01, ***p<0.001, determined with an unpaired Student's *t* test.

On the basis of temporal expression, causal inference of candidate effectors can be tested. A search of cytokine profiles suggested a strong correlation following IFNβ synthesis, further raising the hypothesis for a potential interferon regulatory loop mechanism that is responsible for modulating sterol biosynthesis. First, we investigated directly whether IFNβ is responsible by infecting BMDM from ifnβ−/− mice and examining gene expression for representative members of the sterol pathway. Figure 7A shows that following the genetic ablation of IFNβ, there is still statistically significant sterol gene expression reduction but that there is a partial loss in the degree of reduction indicating that IFNβ is not absolutely necessary. It is possible that other type I IFN members may compensate for the lack of IFNβ. The redundancy among the various type I interferons can be directly evaluated through genetic knockout of their shared receptor, IFNAR1.

For this reason we next investigated whether the sterol response to infection is dependent on the type I interferon receptor. To this end, primary macrophages derived from IFNAR1−/− mice were challenged with mCMV or IFNβ, and the sterol biosynthesis gene expression and free cholesterol levels were analyzed. As shown in Figure 7B–D, the lack of interferon type I receptor abolished the ability of macrophages to reduce both sterol biosynthesis gene expression and cholesterol yield upon either infection with mCMV or treatment with IFNβ. We conclude from these experiments that a type I interferon-dependent innate immune response stringently regulates the metabolic alteration of the sterol biosynthesis network observed upon infection.

**Figure 8:**
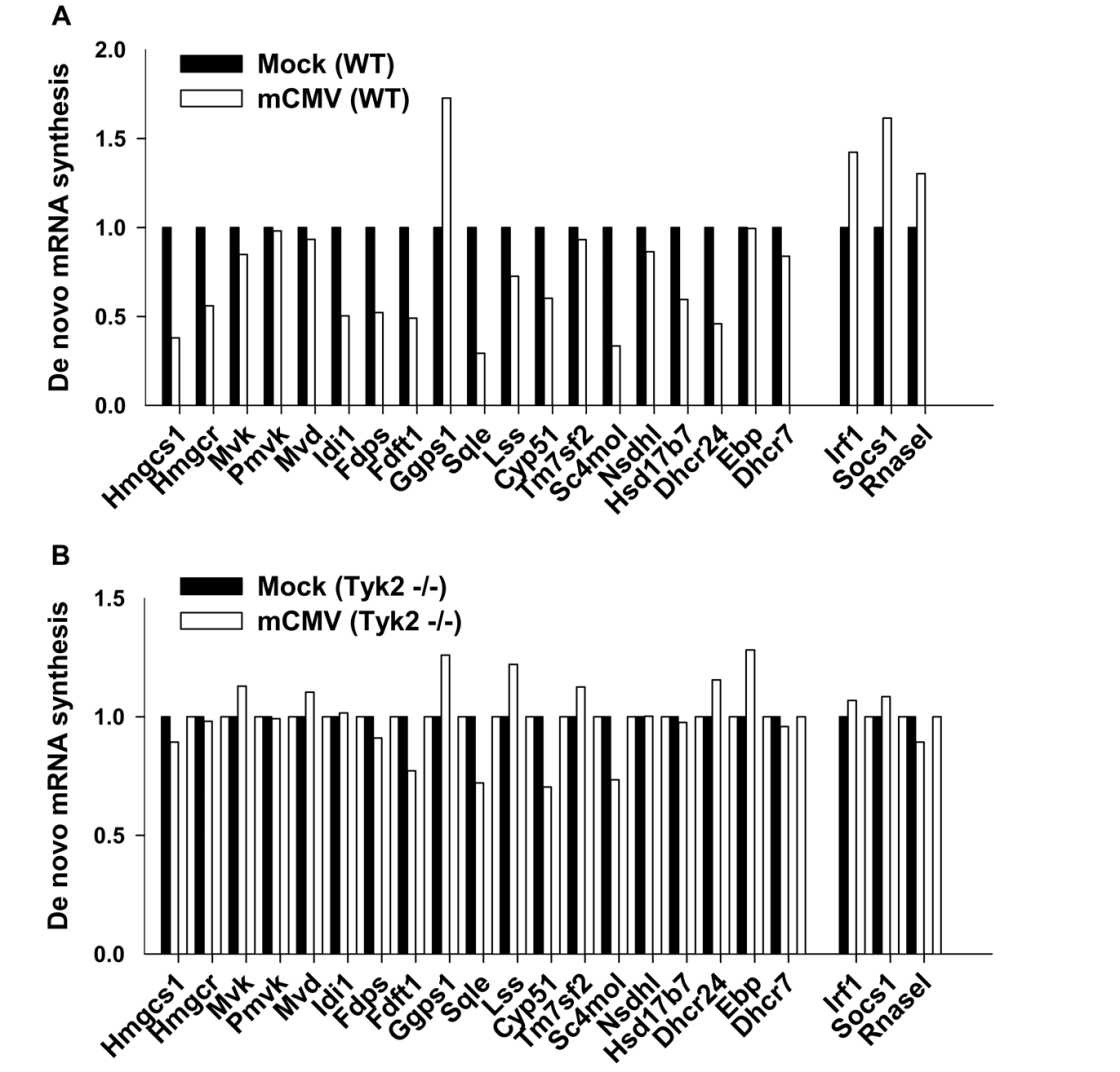
Measurement of de novo mRNA synthesis of sterol biosynthesis genes upon viral infection. Wild Type or Tyk2^−/−^ BMDM were infected with mCMV at an MOI of 1 for 1 h. De novo RNA was labeled between 360 and 390 min post-infection, isolated, and hybridized to Affymetrix Gene 1.0 ST microarrays (Materials and Methods). After scanning and data capture, gene expression in mock-infected or infected cells was analyzed, and for the purposes of presentation, gene expression values from control (mock infected) BMDM (black) were adjusted to a value of 1. Values for expression in infected cells (white) were then expressed as a number relative to the control.

Type 1 interferon has an important role in the control of mCMV replication, and the tyrosine kinase 2 (Tyk 2) signaling component is absolutely essential for the type I defense against mCMV infection. Notably, the lack of Tyk2 is known to selectively impair the transcription of only a subset of virally induced IFNAR1 responsive genes [51]. Since this occurs at the promoter-transcriptional level, we first asked whether the down-regulation of the sterol pathway in response to infection also occurs at the level of gene transcription. For this purpose and to directly measure the level of de novo transcription of members of the sterol pathway, we exploited a recently established labeling protocol for the isolation and analysis of newly transcribed RNA [52]. In these experiments, macrophages were infected with mCMV in the presence of 4-thiouridine, for 30 min at 6 hpi, allowing efficient labeling of nascent RNA for isolation and interrogation by microarray analysis. Figure 8A shows that infection by mCMV results in the anticipated reduced level of newly transcribed RNA of the sterol biosynthetic pathway genes. Next we sought to test whether the Tyk2 receptor-signaling component is required for the type I interferon-dependent down-regulation of the sterol pathway. For these experiments we used tyk2−/− macrophages and observe an almost complete abrogation of the transcriptional down-regulation by mCMV infection (Figure 8B). These results demonstrate a requirement for Tyk2 in the mCMV-mediated gene down-regulation of the sterol biosynthesis pathway and suggest a novel role of interferon type I receptor signaling as a transcriptional modifier of the host's metabolic response to infection.

### SREBP2 Is Down-Regulated upon Infection and IFN Treatment by a Type I Interferon-Dependent Mechanism

**Figure 9:**
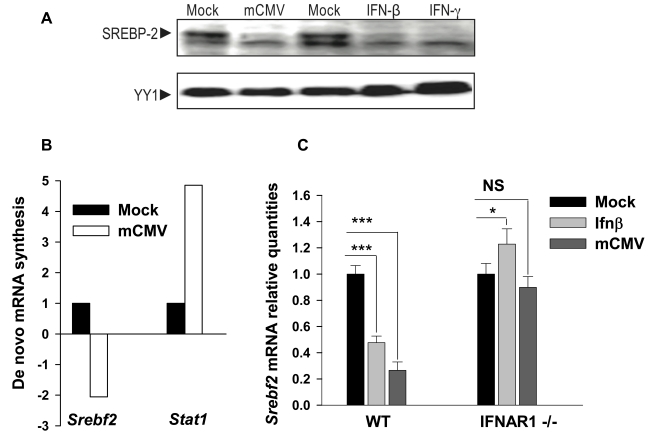
Regulation of SREBP2 by mCMV infection and IFNβ treatment. (A) Comparison of cleaved SREBP2 protein in mock-infected (lane 1), mCMV-infected (MOI of 1) (lane 2), mock-treated (lane 3), IFNβ- (50 U/ml) (lane 4), or IFNγ- (50 U/ml) treated (lane 5) BMDM for 24 h by Western blot analysis using YY1 as a loading control. Arrow indicates SREBP2 cleaved form that is induced upon lovastatin and ezetimibe treatment from liver extracts of cholesterol-fed mice (see Figure S8). The blot is representative of two independent experiments with biological triplicates for each experiment. (B) Wild type BMDM were infected with mCMV for 1 h. De novo RNA was labeled between 360 and 390 min post-infection, isolated, and hybridized to Affymetrix Gene 1.0 ST microarrays (Materials and Methods). After scanning and data capture, gene expression in mock-infected or infected cells was analyzed, and for the purposes of presentation, *Srebf2* gene expression values from control (mock-infected) BMDM (black) were adjusted to a value of 1. Values for expression in infected cells (white) were then expressed as a number relative to the control. (C) BMDM from wild type or IFNAR1−/− knockout mice were treated with 10 U/ml of IFNβ or infected with mCMV. After 24 h, RNA was collected and the gene expression of *Srebf2* was measured by qRT-PCR. Results show the level of gene expression of the treated or infected samples relative to the mock-treated samples. Bars represent the mean ± SD of biological quadruplicates. *p<0.05, **p<0.01, ***p<0.001, determined with an unpaired Student's *t* test.

The above studies strongly point to a transcriptional mechanism in down-regulating the sterol pathway upon infection. The sterol regulatory binding protein 2 (SREBP2) is the principal transcription factor involved in coordinating the regulation of the sterol biosynthesis pathway [53]. SREBP2 is synthesized as a precursor and anchored in the endoplasmic reticulum membrane and through limited proteolysis is activated to generate mature forms that can enter the nucleus and interact with multiple sterol pathway genes to coordinate their expression. Hence, in order to gain further insight into the potential mechanism for participating in the transcriptional down-regulation of the sterol pathway, we investigated in the first instance the protein levels of activated cleaved forms of SREBP2 upon infection and interferon treatment. Accordingly, we next performed Western blot experiments to determine levels of mature form of SREBP2. In these experiments, infection of macrophages with mCMV at 24 hpi developed a significant decrease in the nuclear form (Figure 9A). Furthermore, treatment of macrophages with either IFNβ or IFNγ clearly exhibits a decrease in SREBP2 levels (Figure 9A). We next sought to examine whether this is also seen at the level of transcription. In experiments measuring de novo RNA synthesis, we observed a specific transcriptional reduction from the *Srebf2* gene upon infection while increased levels of transcription are seen for interferon-associated transcription factor Stat1 (Figure 9B), indicating a selective transcriptional basis for the reduced levels of expression. Markedly, the reduction in RNA levels upon infection was completely reversed upon genetic ablation of the *ifnar1* gene (Figure 9C). Altogether these results demonstrate a coordinate reduction in SREBP2 at both the protein and RNA expression level upon infection, which is tightly dependent on activation of the type 1 interferon receptor.

## Discussion

Through the application of a pathway biology strategy integrating genomic, lipidomic, and biochemical approaches with bioinformatics, we show, for the first time, the coupling of the type I interferon response upon viral infection to the sterol pathway and identify the mevalonate-isoprenoid arm as playing a pivotal role in antiviral functions. A definitive link to sterol metabolism that is independent of cholesterol is established by the observation that the anti-viral effect of down-regulating the sterol pathway upon infection is completely blocked if cells are provided with an excess of mevalonate but not cholesterol. Furthermore, the anti-viral potency of type 1 interferon is severely diminished in the presence of excess geranylgeraniol metabolite, highlighting a requirement of the mevalonate-isoprenoid branch as part of an interferon mechanism for protecting against infection. Further on the basis of genetic elimination studies we unequivocally document a molecular dependency between sterol biosynthesis and interferon signaling upon infection, leading to a modest but highly significant coordinate decrease in gene expression, which subsequently causes a marked alteration in the metabolic activity of the sterol pathway.

### A Two-Step Immune-Signaling Cascade Involved in Regulating Sterol Biosynthesis upon Infection

**Figure 10:**
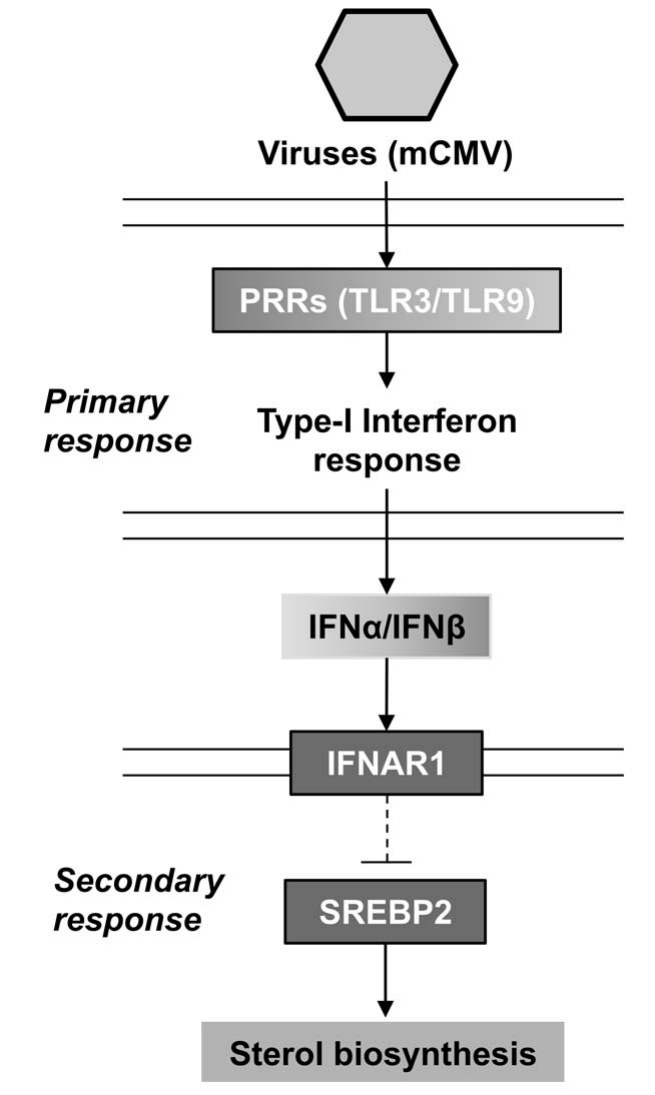
Proposed model for down-regulation of the sterol synthesis by type I interferon response to viral infection.

Our results are consistent with a model involving a two-step interferon response for modulating endogenous sterol pathway activity upon infection. Figure 10 illustrates the two signaling cascades, a virus-induced interferon-producing signal and an interferon receptor-mediated secondary signal. The first is initiated by the detection of virion proteins and nucleic acids by host recognition receptors with the result of the infected cell producing type I interferon. As part of the second step all type I interferons bind to one common receptor (IFNAR1). The IFN-α/β receptor (IFNAR1) signals through the JAK/STAT pathway by phosphorylation of the Janus kinase (JAK)1, tyrosine kinase (Tyk)2, and signal transducer and activator of transcription (STAT)1 and STAT2, which subsequently modulates a diverse array of genes. In the case of mCMV the first step has been extensively investigated and shown to involve TLR2, TLR3, and TLR9 recognition receptors [54],[55], whose activation leads to the induction of transcription factors, NFkB, ATF2/c-Jun, and IRF3 that directly activate IFNα and β genes. Interestingly, previous studies [5],[37] have shown that microbial activation of TLR3 or TLR4 inhibits by an as-yet unknown mechanism LXR target genes such as ABCA1, resulting in the inhibition of cholesterol efflux from macrophages. This is reported to occur in a type I interferon-independent manner [5]. Similar to microbial-mediated TLR activation of IRF3, many viruses including mCMV potently induce IRF3 and may also have the potential to inhibit LXR functions. Despite recent progress in the definition of links between intracellular cholesterol homeostasis and innate immunity, little is known regarding the influence of interferon-regulated signaling on this phenomenon.

In the present study, we demonstrate that transcriptional regulation of the cellular sterol biosynthesis pathway upon infection has an impact on viral replication and depends on an interferon-regulated loop involving type 1 interferon signaling. Specifically, we show that infection of cells by a wide range of viruses or direct interferon stimulation is accompanied by the down-regulation of sterol biosynthesis as a result of reducing the rate of sterol gene transcription. In the context of ligand-activation of the type I receptor, we also demonstrate that this requires the Tyk2 signaling component (Figure 10).

In the context of type I interferon genes induced upon infection, it is worth noting that viperin, a type I interferon-regulated gene, is involved in cellular defense against a number of viruses and functions to disrupt cholesterol-rich lipid rafts that are used as viral production sites in the cell [34],[38],[56]. In addition, an intracellular interaction of viperin with Fdps, an enzyme essential for isoprenoid biosynthesis (Figure 3), has been reported to lower, by a small extent, the activity of the enzyme [38]. It is not known whether targeting Fdps enzyme activity alone is an effective anti-viral mechanism, although the RNAi targeting results of Figure 5 (panel C) suggest that this may be a plausible mechanism (Figure 5C). However, it is more likely that a combination of interferon-mediated transcriptional down-regulation of the sterol biosynthesis genes and the potential enzymatic protein modification at the isoprenoid branch point represents a concerted anti-viral host defense mechanism.

### Coordinate Transcriptional Regulation of Sterol Pathway Members

From a transcriptional perspective, the sterol biosynthesis pathway genes are co-ordinately controlled by the sterol regulatory element binding protein 2 transcription factor (SREBP2). Significantly, we find in our system that the overall abundance of the mature protein (the proteolytically cleaved active form) and the rate of gene transcription of its gene are significantly reduced upon infection or interferon treatment. Significantly, both are strictly dependent on the presence and activation of the type 1 interferon receptor *Ifnar1*. These findings suggest that a possible mechanism for the coordinate down-regulation of sterol biosynthesis is by interferon regulation of *Srebf2*. Interestingly and consistent with the possibility of interferon regulating *Srebf2*, chemical inhibition of SREBP2 has been shown to inhibit HCV replicon activity [32]. This would also support the view of implicating negative feedback on SREBP-2 via oxysterol metabolites. Further studies are required to elucidate more precisely the mechanism or mechanisms by which interferon mediates down-regulation of the sterol biosynthesis pathway. Whatever the mechanism, the IFN-dependent coupling of the mevalonate-sterol metabolic network and anti-viral activity represents a previously unrecognized mechanism in the regulation of protective immunity.

From an immune response and metabolic/pharmacological perspective, modulating cholesterol biosynthesis via small, coordinate transcriptional changes offers advantages and disadvantages over single enzyme control. At the homeostatic level, coordinate control of a metabolic pathway could potentially increase the robustness of modulation; the redundant rate-limiting interactions, downstream of the true rate-limiting interaction, can protect the pathway from surges in the levels of downstream metabolites. Coordinate control also increases the specificity of the pathway modulation as a small reduction of the enzyme level in an interaction ensures that the level of the interacting metabolite need not drop as far to affect a reduction in flux. This has the advantage of potentially lessening the impact on other branched or cross-linked pathways that use the same metabolites and thus provides a high degree of pathway specificity.

### Therapeutic Perspective

Several viruses including human CMV have been reported to be sensitive to statin administration [25],[26],[28]–[31]. Although the mechanism of action of most is not known, it has in some cases been correlated with a lower abundance of cholesterol in lipid rafts of cell membranes. A recognized potential complicating factor of using statins to specifically reduce cholesterol levels is that suppression of the proximal mevalonate arm also perturbs the synthesis of branch derivatives such as geranylgeraniol and farnesol involved in the protein farnesylation and prenylation pathways. In the case of HCV, the mechanisms of the inhibitory effects of the statins have been examined extensively and have been shown to relate to the prenylation of a host protein (FLB2) essential for viral replication [18],[57]. Recently a combination chemical screening study has been conducted to explore how the sterol and protein prenylation pathways work together to affect HCV in a replicon assay [32]. In agreement with those studies we also find reduced mCMV growth in siRNA knock-down experiments targeting enzymes in the isoprenod biosynthesis pathway. These studies indicate the importance of the geranylgeranylation to viral replication. Although, it is worth noting that the isoprenoid biosynthesis pathway is highly complicated with multiple branch points involving redundant enzymatic steps, sharing of subunits, and competing reactions. In our current study, we uncoupled the cholesterol synthesis pathway from non-steroidal modifications through targeted metabolic rescue and siRNA knock-down studies of mCMV and reveal an absolute requirement for the prenylation branch of the sterol pathway for mediating anti-viral effects. As further indicated from computational modeling work (unpublished data), targeting HMGCR is likely to have a broad range of non-specific effects on various efferent branch points of the pathway and thus may well not be ideal for anti-infective therapy. In addition, statins are also known to have a range of immune-modulatory activities by mechanisms yet to be fully characterized. In this context, it is worth noting that the activity of the type I interferons, especially IFNβ, have considerable overlap with many of the immune-related activities of statins [58]. Moreover, it is especially noteworthy that IFNβ treatment in patients has also been reported to have decreased plasma cholesterol levels [59],[60]. Since our studies uncover a molecular dependency of type 1 signaling, including a Tyk2 signaling component, this may provide an entirely new therapeutic pathway for lowering cholesterol. Moreover, our findings may have important implications for the development of broadly active new adjuvant strategies (e.g., the use of inhibitors of SREBP2 activity) to existing anti-infective therapies (e.g., antiviral drugs such as ganciclovir). On this basis we posit the principal of using metabolic modifiers, i.e. drugs that target metabolic pathways, of protective innate immunity as holding future promise for developing host-directed anti-viral therapies. Overall, this study supports the original concept [40],[41] of selectively targeting host pathways as an efficacious anti-infective strategy.

## Materials and Methods

### Microarray Experiment, Bioinformatic Analysis

Microarray analysis of the time course experiments of infected and interferon treated macrophages were conducted using Agilent microarray platform and a detailed description of the experimental set up; statistical and bioinformatics analysis is in the Supporting Information section. All other microarray studies were conducted using Affymetrix (Mouse Genome 430) microarray platform. Data from hybridized Affymetrix microarrays were acquired using proprietary Affymetrix platform scanners and GCOS software (Affymetrix). Processed CEL files were imported into Partek Genomics SuiteTM (MO, USA), then background corrected, quantile normalized, and probe-set summarized using the RMA algorithm [61],[62]. A non-specific filter was applied to remove genes that were not expressed on any of the samples across the experiment. Microarray signals were then per-gene normalized to the average of the three mock samples (which was set to a value of 1) for visualization purposes in the heat map for Figure 6. In the case of de novo RNA expression, analysis was performed using the Affymetrix Mouse Gene 1.0 ST arrays, consisting of a total of eight chips and three experiment factors: time (60–90 min, 360–390 min), genetic background (Tyk2KO, WT), and treatment (mock, mCMV). Data from hybridized chips were acquired using GCOS software (Affymetrix). Prior to further processing and analysis with the R statistical programming environment, Affymetrix Power Tools (APT, Affymetrix) were used to summaries and annotate chip data to gene level. After initial quality control assessment, data were background-corrected, quantile normalized, and probe-set summarized using the RMA algorithm.

### Mice BMDM Cultures

Wild type C57BL/6 and BALBc were from the Biomedical Research Resources, Little France, University of Edinburgh. IFNβ−/− and Tyk2−/− mice were from the Institute of Animal Breeding and Genetics Veterinary University of Vienna. BMDM were derived from monocytes obtained from femurs of male mice aged 10 to 12 wk. Cells were grown in DMEM-F12 media supplemented with 10% L929 cell-conditioned medium as a source of macrophage colony-stimulating factor (M-CSF) for 7 d as described [63]. Characterization of BMDM was performed by standard flow cytometry, evaluating the presence of the F4/80 marker and CD11b surface protein. In average of all experiments more than 93% of cells possessed both proteins.

### Viruses

The mouse CMV C3X strain, generated from the recombinant C3X bacterial artificial chromosome clone and originally derived for the Smith strain of mCMV [64], was propagated in NIH 3T3 cells, and titers were determined by standard plaque assay on MEFp53−/−. For live cell assay, NIH/3T3s were infected with a recombinant mCMV expressing the green fluorescent protein (GFP) marker inserted in front of the ie2 gene (pSM3fr-rev, called mCMV-GFP in this study [65]). Viral growth curves comparing wild type and GFP virus were assessed by standard plaque assay, and the results showed no differences between the growth curve of the two viruses (unpublished data). To establish the role of viral gene expression in the regulation of sterol genes, the mCMVdie3 strain was used [50]. For the microarray experiment, Semliki Forest Virus (SFV, MOI of 10), Herpes simplex virus type 1 (HSV1, MOI of 1), Vaccinia virus (VV, MOI of 1), and Adenovirus (Ad, MOI of 100) were used to infect BMDM for 1 h in DMEM:F12 3% FCS, 10% L929, and 100 U of penicillin/streptomycin per ml.

### Infection

BMDM and NIH/3T3 were infected with the different viruses at an MOI of 1, unless specified. For BMDM, viral stock was diluted in DMEM:F12 3% FCS, 10% L929, and 100 U of penicillin/streptomycin per ml, and after 1 h adsorption, cells were washed in PBS and incubated in fresh DMEM:F12 10% FCS, 10% L929, and 100 U of penicillin/streptomycin per ml. For NIH/3T3 viral stock was diluted in DMEM 3% CS and 100 U of penicillin/streptomycin per ml, and after 1 h adsorption, cells were washed in PBS and incubated in fresh DMEM:F12 10% CS and 100 U of penicillin/streptomycin per ml. SFV (MOI of 10), HSV1 (MOI  = 1), VV (MOI of 1), and Ad (MOI of 100) were used to infect BMDM for 1 h in DMEM:F12 3% FCS, 10% L929, and 100 U of penicillin/streptomycin per ml.

### Cytokines and Pharmacological Treatments

IFNγ (Boehringer Manheim Corp), IFNβ, IL6, TNF, and IL1β (Biosource International, USA) stock were dissolved in PBS supplemented with 0.2% BSA and diluted in fresh media just prior to the experiment. The effect of cytokine treatment on cell viability was tested for each concentration used in the experiment and did not show any alteration of viability. For the pharmacological experiment, 25 mg of simvastatin (Sigma-Aldrich) was activated by hydrolysis of the lactone by adding 1 ml of 0.1 N NaOH, 100% ethanol. After heating at 50°C for 2 h, the solution was neutralized with HCl to a pH of ≈7.2 and sterilized by filtration through a 0.2 µm filter. The stock solution was diluted to the appropriate concentration in sterile PBS and the solution was aliquoted, stored at −20°C, and used within a month of activation. Mevalonate and water soluble cholesterol (Sigma-Aldrich, Germany) was resuspended in media to the appropriate concentration and sterilized by filtration through a 0.2 µm filter. Geranylgeraniol and farnesol squalene (Sigma-Aldrich, Germany) stocks were dissolved in DMSO and sterilized by filtration through a 0.2 µm filter. The stock solutions were dissolved in media at the appropriate concentration just prior to the experiment. The final concentration of DMSO in media did not exceed 0.1%. Effects of sterol intermediates treatment on the cell were tested for each concentration used in the experiment and did not show any alteration of viability. Gancyclovir (Cymevene, Hoffman-La Roche, UK) was resuspended in saline solution and sterilized by filtration through a 0.2 µm filter. Gancyclovir was then diluted in media, to the indicated concentration.

### Quantitative RT-PCR

Taqman Primer probe sets were purchased from Applied Biosystems, Warrington, UK (Assay ID: Hmgcs1: Mm00524111-m1; Hmgcr: Mm01282499-m1; Idi1: Mm00836417-g1; Sqle: Mm00436772-A1). For each sample QRT-PCR was performed in 20 µl volumes using MicroAmp Optical 96-well reaction plates and MicroAmp Optical Caps (Applied Biosystems). Two microliters of diluted RNA samples (≈100 ng of RNA) were added to 10 µl of 2× PCR master mix, 1 µl of a Taqman primer/probe set (Applied Biosystems, CA) for the gene of interest at the recommended concentration, 0.25 µl of Superscript III (Applied Biosystems, CA), and 6.25 µl of double-distilled H_2_0. After an initial incubation at 50°C for 30 s to activate the RNA polymerase, samples were then subject to 40 cycles under Taqman standard conditions (combined annealing and primer extension phase at 60°C for 1 min and a short denaturation at 72°C for 30 s). Stratagene MXPro software was then used to analyze the data. Threshold determinations were automatically performed by the instrument for each reaction. The C_T_ values were exported into Microsoft Excel and relative quantification of marker gene mRNA expression was calculated with the comparative C_T_ method [66].

### Western Blot Analysis

BMDM cells were washed with PBS and resuspended in whole-cell lysis buffer (50 mM Tris-HCl, pH 7.5, 100 mM NaCl, 1% NP40, protease inhibitors, and phosphatase inhibitors), and cell lysates were centrifuged at 4°C for 10 min and the collected supernatants were stored at −20°C. Protein concentration was measured by Pierce BCA assay (Thermo Scientific). For Western blotting, proteins were separated by SDS-PAGE, transferred to Immobilon-FL membranes (Millipore), and probed with goat anti-HMGCR (Santa Cruz, sc-27578, 1∶500), goat anti-SQLE (Santa Cruz, sc-49754, 1∶500), anti-HMGCS1 (Santa Cruz, Sc-32422, 1∶500), mouse anti-mCMV IE1 (Chroma 101, 1∶1000), and rabbit anti-β-actin (Cell Signalling, 4970, 1∶2500) diluted in PBST (0.1% Tween20). For secondary anti-goat IR-680 (Invitrogen, A21088, 1∶10,000), IR-800 anti-mouse (Thermo Fisher Scientific, 35571, 1∶10,000), and IR-800 anti-rabbit (Cell Signalling, 5151, 1∶10,000), antibodies were diluted in Odyssey blocking buffer (0.1% Tween20, 0.01% SDS). For probing, visualization, and quantification, the Odyssey protocol (LI-COR) was followed. The fluorescence was quantified by Odyssey system (Li-COR). For details of anti-mouse SREBP-2 polyclonal antibody (custom antibody raised against mature SREBP-2 form [67]) and immunoblot procedures, see Text S1.

### Measurement of Free Cholesterol Concentration by Enzymatic Assay

Intracellular cholesterol concentration was determined enzymatically using the Amplex-Red cholesterol Assay Kit (Molecular Probes) according to manufacturer recommendations. Briefly, cells were washed with 1 ml ice-cold PBS and then lysed in 200 µl cold Lipid buffer containing 0.5 M of potassium phosphate, pH 7.4, 0.25 mM cholic acid, and 0.5% triton X-100. Cell lysates were sonicated on ice with three 10-s pulses at high intensity. 20 µl were then used to determine protein concentration using a standard BCA assay to normalize the protein concentration. For cholesterol measurement, 20 µl of each sample were added to the 80 µl assay solution, which contained 300 µM Amplex Red reagent, 2 U per ml HRP and 2 U per ml cholesterol oxidase, 0.1 M of potassium phosphate, pH 7.4, 0.05 mM cholic acid, and 0.1% triton X-100. After preincubation for 30 min at 37°C under light exclusion conditions, fluorescence was measured using excitation at 530±2.5 nm and fluorescence detection at 590±2.5 nm with a Polarstar Optima Multifunciton Microplate Reader (BMG Labtech, UK). The values were corrected from the background. The relative amount of free cholesterol to the mock-treated samples was calculated using the manufacturer's supplied standard curve.

### Analysis of Lipids Using High-Performance Liquid Chromatography/Mass Spectrometry and Electrospray Ionization

An Agilent high-performance liquid chromatography (HPLC) system coupled with an Applied Biosystem Triple Quadrupole/Ion Trap mass spectrometer (4000Qtrap) was used for quantification of individual polar lipids (Phospholipids and sphingolipids). Electrospray ionization-based multiple reaction monitoring (MRM) transitions were set up for the quantitative analysis of various polar lipids [68]. HPLC atmosphere chemical ionization/MS were carried out for analysis of sterols [69].

### Live Cell Replication Assay

To measure the effect of multiple drugs and siRNA transfection on viral growth, a sensitive live cell infection assay was developed using the properties of the mCMV GFP tagged virus. 1.5×10^4^ NIH/3T3 cells were infected for 1 h in black 96-well plates (Costar, UK) at an MOI of 0.2 in 25 µl of fresh DMEM phenol red-free media, 3% CS, and 100 U of penicillin/streptomycin per ml. After infection, the inoculums were carefully removed by pipetting and replaced by 150 µl of DMEM phenol red-free media with 10% FCS. Viral growth was measured by recording the GFP signal over time using an OPTIMA Polarstar plate reader (excitation wave length of 485 nm and emission of 520 nm). As an optimization step we checked the correlation between GFP levels and MOIs. Results showed a good correlation between multiplicity of infection and growth kinetics (Figure S4). Comparing the GFP value and number of viral particles per ml using plaque assay checked levels of GFP signal corresponding to different levels of virus. Results (unpublished data) showed a strong correlation between differences in levels of GFP expression and differences in number of viral particles assessed by plaque assay: a drop of 20% of GFP signals corresponding to a log difference in the number of viral particles monitored by plaque assay.

### Transfection and siRNA Knock-Down Assays

For transfection, siRNA (SMARTpools-ON-TARGETplus modification) from Thermo Fisher Inc. were purchased. The samples were supplied at a concentration of 5 mM and diluted and aliquoted in 2 µM amounts. To transfect at a final concentration of 20 nM per well, 1 µl of siRNA SMARTpool was used with 9 µl of Optimem (Invitrogen, CA, USA) solution while 0.4 µl of Dharmafect 1 (Dharmacon, Perbio Science, Bonn, Germany) was mixed with 9.6 µl Optimem. Following incubation for 5 min, the siRNA mix was added to the Dharmafect 1 (0.4%) mix and incubated for a further 30 min, after which 1.5×10^4^ NIH3T3 cells in 80 µl of DMEM 10% CS medium lacking antibiotics was added to the siRNA:Dharmafect 1 complexes. Growth medium was removed and cells were washed 1× in PBS before 100 µl of the siRNA: Dharmafect 1 liposomes were added. Transfection conditions were optimized by using siGLO RED from Thermo Fisher Scientific (Dharmacon) as an indicator of transfection efficiency and cell viability was assessed as described before. For every gene targeted, the knock-down efficiency was checked by QPCR after 48 h incubation. Each of the three genes targeted (*Hmgcs1*, *Hgmcr*, and *Idi1*) were knocked down by more than 70%, 48 h after transfection (Figure S5). Knock-down efficiency and cell viability were also checked at 5 d post-infection for the mCMV-GFP assay, and showed no alteration of the viability and a knock-down efficiency ≥50% (unpublished data).

### Nascent RNA Expression Profiling of TYK2−/− mCMV Infected Macrophages Experiment

BMDM were isolated and grown in the presence of Csf1 derived from L929 cells as described [63] except cells were cultivated in 15 cm diameter tissue culture plates for 7 d before treatment. Incorporation of 4-thiouridine (Sigma) into nascent RNA was undertaken as described [52]. In brief, at 360 min post-infection, 10 ml medium was aspirated from all plates, added to 80 µl 4-Thiouridine, mixed, and immediately returned to the culture dish. After 30 min, to end the RNA labeling period, terminate transcription, and lyse the cells, medium was aspirated from the labeled BMDM and replaced with 4 ml of RLT lysis buffer (Qiagen). Total RNA was isolated using an RNeasy Midi kit (Qiagen) according to the manufacturer's instructions, quantitated using a Nanodrop (Thermo Scientific), and integrity was confirmed using an Agilent Bioanalyser (Agilent UK). Newly transcribed RNA (ntRNA) was then isolated as described in [52] and again quantitated using a Nanodrop.

Processing of ntRNA samples (94 ng) for hybridization to Affymetrix Mouse Gene 1.0 ST arrays was undertaken according to the manufacturer's instructions (Affymetrix). Hybridisation, washing, staining, and scanning of the arrays were also undertaken following standard Affymetrix protocols. For the purposes of presentation, gene expression values for the specific genes of interest from control (mock-infected) BMDM were adjusted to a value of 1. Values for expression in infected cells (white) were then expressed as a number relative to the control.

### In Vivo Studies

All animal experiments had approval by the local animal ethics committee (University of Edinburgh, Edinburgh, UK) in accordance with recommendations of the Federation of European Animal Science Association and European legislation. Twelve mice (C57/BL6, Charles River, 12 wk of age) were randomized into two groups of six animals each in two separate experiments. Simvastatin was prepared as described above. The dosages of statins used in the present investigation were chosen according to the literature [30]. At day 1, mice were inoculated i.p. with 2×10^6^ PFU per mouse. Animals were sacrificed 4 d post-infection. Spleen, liver, kidney, heart, and lung were harvested and sonicated as a 10% (wt/vol) tissue homogenate, and titers were determined by standard plaque assays, including centrifugal enhancement of infectivity on MEFp53−/−. The dashed line indicates the limit of detection (5×10^2^ PFU/g). Horizontal bars indicate the median values.

### Statistical Analysis

Normalization, filtering, statistical hypothesis testing for microarray data was carried out within the R Language and Environment for Statistical Computing (www.r-project.org), using packages provided through the Bioconductor repository (www.bioconductor.org). The majority of explorative analyses and visualizations were conducted with Partek (Partek Incorporated, USA) and GeneSpring GX (Agilent). Statistical analyses on other data sources were performed in Microsoft Excel software. For real-time PCR and replication assay, all graphs represent the mean ± SD. An unpaired Student's *t* test was used for evaluation of statistical significance of real-time PCR. For in vivo experiment a Mann-Whitney U test was used. See Text S1 for statistical analysis of microarray experiments. Statistical significance: *p<0.05, **p<0.01, ***p<0.001.

## Supporting Information

Figure S1 Pie chart representing the percentage of lipid class present in (A): 62 down-regulated and 133 up-regulated Lipid Associated Genes (LAGs) upon mCMV infection, (B) 51 down-regulated and 65 up-regulated LAGs after IFNγ treatment, and (C) 35 down-regulated and 47 up-regulated LAGs by mCMV infection and IFNγ treatment.
(3.83 MB TIF)

Figure S2 Concentrations of four cholesterol synthesis-related metabolites following mCMV infection. (A–D) Concentrations of Zymosterol, 14-demethyl-lanosterol, 7-dehydro-cholesterol, and free cholesterol are measured from lipid extract by MRM analysis (Materials and Methods) from BMDM following mCMV infection (MOI of 1 at 24 and 48 hpi). Bars represent means ± SD of triplicates biological measurements.
(0.31 MB TIF)

Figure S3 Lipidomic analysis using high performance liquid chromatography/mass spectrometry and electroscopy ionization of mCMV infected BMDM. Lipid analysis of total glycerophospholipids (phosphatidylcholine, phosphatidylserine, phosphatidylethanolamine; A) and individual species of phosphatidylcholine (B) and phosphatidylserine (C) (Materials and Methods). Bars represent means ± SD of triplicates biological measurements.
(6.02 MB TIF)

Figure S4 Live cell mCMV-GFP growth curve. NIH3T3 were infected at an MOI of 0.01, 0.1, and 1 with mCMV-GFP for 1 h, and viral inoculums were replaced by fresh media. GFP signal was measured at a different time post-infection (Materials and Methods).
(0.09 MB TIF)

Figure S5 Knock-down efficiency. NIH3T3 cells were transfected with 0.4% Dharmafect 1 and 25 nm of Hmgcs1, Hmgcr, or Idi1 SiRNA smart pool (Dharmacon). After 48 h, RNA was collected and QPCR was performed to check gene expression for Hmgcs1, Hmgcr, and idi1. Gapdh was used for normalization. Hmgcs1 shows an 81% decrease in expression following transfection, Hmgcr a 70% decrease, and Idi1 an 82% decrease. Bars represent means ± SD of triplicate biological measurements.
(1.23 MB TIF)

Figure S6 mCMV infection reduced free intra-cellular cholesterol in a dose-dependent manner in BMDM at 48 hpi. BMDM were infected at different MOI (0, 0.1, 0.2, 0.5, and 1) with mCMV virus. Data are represented as the percentage of free intracellular cholesterol concentration from infected cells in comparison to mock treatment. Results represent means ± SD from two independent experiments with triplicate biological measurements for each experiment.
(1.32 MB TIF)

Figure S7 Effects of various concentrations of Simvastatin on NIH-3T3 fibroblasts cell viability. NIH-3T3 fibroblasts were treated with various concentrations of Simvastatin or with vehicle for 72 h. Cell viability was determined using the Cell titre blue assay as described in Methods. Cell viability is expressed as the percentage of fluorescence signal from treated cells compared to untreated cells. Graphs represent the average values (±SD) of two independent experiments with triplicate biological measurements for each experiment.
(0.04 MB TIF)

Figure S8 Specificity of the SREBP2 antibody. Lane 1 and 2: Nuclear extract protein prepared from livers of mice fed chow supplemented with either a 2% cholesterol diet (CHOL) or a mixture of lovastatin and ezetimibe were loaded as controls. Arrow indicates the specific SREBP2 cleaved form. As a comparison, lanes 3 and 4 show nuclear protein extracts prepared from macrophages cultured from mock or mCMV infected. YY1 protein was used as a loading control. (1.36 MB TIF)

Table S1 List of lipogenic associated genes (LAGs) down- (A) or up- (B) regulated by mCMV infection or down- (C) and up- (D) regulated by IFNγ treatment.
(0.09 MB XLS)

Table S2 Canonical pathway analysis. Analysis was performed using IPA from Ingenuity (www.ingenuity.com): (A) 62 down-regulated LAGs in response to mCMV infection, (B) 133 up-regulated LAGs in response to mCMV infection, (C) 51 down-regulated LAGs in response to IFNγ treatment, (D) 65 up-regulated LAGs in response to IFNγ treatment, (E) 35 down-regulated LAGS in response to mCMV infection and IFNγ treatment, and (F) 47 up-regulated LAGs in response to mCMV infection and IFNγ treatment. For each table, the top 5 most significant pathways are represented.
(0.02 MB XLS)

Text S1 Supporting methods. This file gives an overview of the methods used in this article.
(0.13 MB DOC)

## Abbreviations

CMV: cytomegalovirus
Fdps: farnesyl diphosphate synthase
HCV: Hepatitis C virus
hpi: hours post-infection
HSV1: herpes simplex virus 1
IFN: interferon
IFNAR1: IFN-α/β receptor
JAK: Janus kinase
LAG: Lipid Associated Gene
mCMV: murine cytomegalovirus
MRM: multiple reaction monitoring
PRR: host recognition receptor
SREBP2: sterol regulatory binding protein 2
STAT: signal transducer and activator of transcription
Tyk 2: tyrosine kinase 2

## References

[1] Makowski L, Hotamisligil G. S (2005). The role of fatty acid binding proteins in metabolic syndrome and atherosclerosis.. Curr Opin Lipidol.

[2] Lusis A. J (2000). Atherosclerosis.. Nature.

[3] Wood D. A, Butler S, Riemersma R. A, Thomson M, Oliver M. F (1984). Adipose tissue and platelet fatty acids and coronary heart disease in Scottish men.. Lancet.

[4] Riemersma R. A, Perkins D, Brown A. J, Brown J (1994). Linoleic acid and coronary artery disease.. Am J Clin Nutr.

[5] Castrillo A, Joseph S. B, Vaidya S. A, Haberland M, Fogelman A. M (2003). Crosstalk between LXR and toll-like receptor signaling mediates bacterial and viral antagonism of cholesterol metabolism.. Mol Cell.

[6] Zelcer N, Tontonoz P (2006). Liver X receptors as integrators of metabolic and inflammatory signaling.. J Clin Invest.

[7] Ogawa S, Lozach J, Benner C, Pascual G, Tangirala R. K (2005). Molecular determinants of crosstalk between nuclear receptors and toll-like receptors.. Cell.

[8] Wang S, Wu D, Lamon-Fava S, Matthan N. R, Honda K. L (2009). In vitro fatty acid enrichment of macrophages alters inflammatory response and net cholesterol accumulation.. Br J Nutr.

[9] Yvan-Charvet L, Wang N, Tall A. R (2010). Role of HDL, ABCA1, and ABCG1 transporters in cholesterol efflux and immune responses.. Arterioscler Thromb Vasc Biol.

[10] Bauman D. R, Bitmansour A. D, McDonald J. G, Thompson B. M, Liang G (2009). 25-Hydroxycholesterol secreted by macrophages in response to Toll-like receptor activation suppresses immunoglobulin A production.. Proc Natl Acad Sci U S A.

[11] Zhu X, Lee J. Y, Timmins J. M, Brown J. M, Boudyguina E (2008). Increased cellular free cholesterol in macrophage-specific Abca1 knock-out mice enhances pro-inflammatory response of macrophages.. J Biol Chem.

[12] Haas M. J, Mooradian A. D (2010). Regulation of high-density lipoprotein by inflammatory cytokines: establishing links between immune dysfunction and cardiovascular disease.. Diabetes Metab Res Rev.

[13] Maitra U, Parks J. S, Li L (2009). An innate immunity signaling process suppresses macrophage ABCA1 expression through IRAK-1-mediated downregulation of retinoic acid receptor alpha and NFATc2.. Mol Cell Biol.

[14] Gerbod-Giannone M. C, Li Y, Holleboom A, Han S, Hsu L. C (2006). TNFalpha induces ABCA1 through NF-kappaB in macrophages and in phagocytes ingesting apoptotic cells.. Proc Natl Acad Sci U S A.

[15] Eguchi J, Yan Q. W, Schones D. E, Kamal M, Hsu C. H (2008). Interferon regulatory factors are transcriptional regulators of adipogenesis.. Cell Metab.

[16] Munger J, Bennett B. D, Parikh A, Feng X. J, McArdle J (2008). Systems-level metabolic flux profiling identifies fatty acid synthesis as a target for antiviral therapy.. Nat Biotechnol.

[17] Kapadia S. B, Barth H, Baumert T, McKeating J. A, Chisari F. V (2007). Initiation of hepatitis C virus infection is dependent on cholesterol and cooperativity between CD81 and scavenger receptor B type I.. J Virol.

[18] Wang C, Gale M, Keller B. C, Huang H, Brown M. S (2005). Identification of FBL2 as a geranylgeranylated cellular protein required for hepatitis C virus RNA replication.. Mol Cell.

[19] Ye J, Wang C, Sumpter R, Brown M. S, Goldstein J. L (2003). Disruption of hepatitis C virus RNA replication through inhibition of host protein geranylgeranylation.. Proc Natl Acad Sci U S A.

[20] Park C. Y, Jun H. J, Wakita T, Cheong J. H, Hwang S. B (2009). Hepatitis C virus nonstructural 4B protein modulates sterol regulatory element-binding protein signaling via the AKT pathway.. J Biol Chem.

[21] Robinzon S, Dafa-Berger A, Dyer M. D, Paeper B, Proll S. C (2009). Impaired cholesterol biosynthesis in a neuronal cell line persistently infected with measles virus.. J Virol.

[22] Zheng Y. H, Plemenitas A, Fielding C. J, Peterlin B. M (2003). Nef increases the synthesis of and transports cholesterol to lipid rafts and HIV-1 progeny virions.. Proc Natl Acad Sci U S A.

[23] Mackenzie J. M, Khromykh A. A, Parton R. G (2007). Cholesterol manipulation by West Nile virus perturbs the cellular immune response.. Cell Host Microbe.

[24] Rothwell C, Lebreton A, Young Ng C, Lim J. Y, Liu W (2009). Cholesterol biosynthesis modulation regulates dengue viral replication.. Virology.

[25] Mohan K. V, Muller J, Atreya C. D (2008). Defective rotavirus particle assembly in lovastatin-treated MA104 cells.. Arch Virol.

[26] Bader T, Fazili J, Madhoun M, Aston C, Hughes D (2008). Fluvastatin inhibits hepatitis C replication in humans.. Am J Gastroenterol.

[27] Liu S, Rodriguez A. V, Tosteson M. T (2006). Role of simvastatin and methyl-beta-cyclodextrin [corrected] on inhibition of poliovirus infection.. Biochem Biophys Res Commun.

[28] Cohen J. I (2005). HMG CoA reductase inhibitors (statins) to treat Epstein-Barr virus-driven lymphoma.. Br J Cancer.

[29] del Real G, Jimenez-Baranda S, Mira E, Lacalle R. A, Lucas P (2004). Statins inhibit HIV-1 infection by down-regulating Rho activity.. J Exp Med.

[30] Gower T. L, Graham B. S (2001). Antiviral activity of lovastatin against respiratory syncytial virus in vivo and in vitro.. Antimicrob Agents Chemother.

[31] Potena L, Frascaroli G, Grigioni F, Lazzarotto T, Magnani G (2004). Hydroxymethyl-glutaryl coenzyme a reductase inhibition limits cytomegalovirus infection in human endothelial cells.. Circulation.

[32] Owens C. M, Mawhinney C, Grenier J. M, Altmeyer R, Lee M. S Chemical combinations elucidate pathway interactions and regulation relevant to Hepatitis C replication.. Mol Syst Biol.

[33] Hong C, Tontonoz P (2008). Coordination of inflammation and metabolism by PPAR and LXR nuclear receptors.. Curr Opin Genet Dev.

[34] Hansson G. K (2005). Inflammation, atherosclerosis, and coronary artery disease.. N Engl J Med.

[35] Chawla A, Boisvert W. A, Lee C. H, Laffitte B. A, Barak Y (2001). A PPAR gamma-LXR-ABCA1 pathway in macrophages is involved in cholesterol efflux and atherogenesis.. Mol Cell.

[36] Diamond D. L, Syder A. J, Jacobs J. M, Sorensen C. M, Walters K. A Temporal proteome and lipidome profiles reveal hepatitis C virus-associated reprogramming of hepatocellular metabolism and bioenergetics.. PLoS Pathog.

[37] Joseph S. B, Bradley M. N, Castrillo A, Bruhn K. W, Mak P. A (2004). LXR-dependent gene expression is important for macrophage survival and the innate immune response.. Cell.

[38] Wang X, Hinson E. R, Cresswell P (2007). The interferon-inducible protein viperin inhibits influenza virus release by perturbing lipid rafts.. Cell Host Microbe.

[39] Hinson E. R, Cresswell P (2009). The antiviral protein, viperin, localizes to lipid droplets via its N-terminal amphipathic alpha-helix.. Proc Natl Acad Sci U S A.

[40] Ghazal P, Gonzalez Armas J. C, Garcia-Ramirez J. J, Kurz S, Angulo A (2000). Viruses: hostages to the cell.. Virology.

[41] Fruh K, Simmen K, Luukkonen B. G, Bell Y. C, Ghazal P (2001). Virogenomics: a novel approach to antiviral drug discovery.. Drug Discov Today.

[42] Wenk M. R (2006). Lipidomics of host-pathogen interactions.. FEBS Lett.

[43] Reddehase M. J, Podlech J, Grzimek N. K (2002). Mouse models of cytomegalovirus latency: overview.. J Clin Virol.

[44] Brautigam A. R, Dutko F. J, Olding L. B, Oldstone M. B (1979). Pathogenesis of murine cytomegalovirus infection: the macrophage as a permissive cell for cytomegalovirus infection, replication and latency.. J Gen Virol.

[45] Selgrade M. K, Osborn J. E (1974). Role of macrophages in resistance to murine cytomegalovirus.. Infect Immun.

[46] Davies P (1976). Essential role of macrophages in chronic inflammatory processes.. Schweiz Med Wochenschr.

[47] Valyi-Nagy T, Bandi Z, Boldogh I, Albrecht T (1988). Hydrolysis of inositol lipids: an early signal of human cytomegalovirus infection.. Arch Virol.

[48] Ishida F, Sato A, Iizuka Y, Kamei T (1989). Inhibition of acyl coenzyme A: cholesterol acyltransferase by 3-hydroxy-3-methylglutaryl coenzyme A reductase inhibitors.. Chem Pharm Bull (Tokyo).

[49] Goldstein J. L, Brown M. S (1990). Regulation of the mevalonate pathway.. Nature.

[50] Ghazal P, Messerle M, Osborn K, Angulo A (2003). An essential role of the enhancer for murine cytomegalovirus in vivo growth and pathogenesis.. J Virol.

[51] Strobl B, Bubic I, Bruns U, Steinborn R, Lajko R (2005). Novel functions of tyrosine kinase 2 in the antiviral defense against murine cytomegalovirus.. J Immunol.

[52] Dolken L, Ruzsics Z, Radle B, Friedel C. C, Zimmer R (2008). High-resolution gene expression profiling for simultaneous kinetic parameter analysis of RNA synthesis and decay.. RNA.

[53] Brown M. S, Goldstein J. L (1997). The SREBP pathway: regulation of cholesterol metabolism by proteolysis of a membrane-bound transcription factor.. Cell.

[54] Tabeta K, Georgel P, Janssen E, Du X, Hoebe K (2004). Toll-like receptors 9 and 3 as essential components of innate immune defense against mouse cytomegalovirus infection.. Proc Natl Acad Sci U S A.

[55] Compton T, Kurt-Jones E. A, Boehme K. W, Belko J, Latz E (2003). Human cytomegalovirus activates inflammatory cytokine responses via CD14 and Toll-like receptor 2.. J Virol.

[56] Jiang D, Weidner J. M, Qing M, Pan X. B, Guo H Identification of five interferon-induced cellular proteins that inhibit west nile virus and dengue virus infections.. J Virol.

[57] Kapadia S. B, Chisari F. V (2005). Hepatitis C virus RNA replication is regulated by host geranylgeranylation and fatty acids.. Proc Natl Acad Sci U S A.

[58] Neuhaus O, Stuve O, Archelos J. J, Hartung H. P (2005). Putative mechanisms of action of statins in multiple sclerosis–comparison to interferon-beta and glatiramer acetate.. J Neurol Sci.

[59] Dixon R. M, Borden E. C, Keim N. L, Anderson S, Spennetta T. L (1984). Decreases in serum high-density-lipoprotein cholesterol and total cholesterol resulting from naturally produced and recombinant DNA-derived leukocyte interferons.. Metabolism.

[60] Morra V. B, Coppola G, Orefice G, De Michele G, Vacca G (2004). Interferon-beta treatment decreases cholesterol plasma levels in multiple sclerosis patients.. Neurology.

[61] Irizarry R. A, Hobbs B, Collin F, Beazer-Barclay Y. D, Antonellis K. J (2003). Exploration, normalization, and summaries of high density oligonucleotide array probe level data.. Biostatistics.

[62] Irizarry R. A, Bolstad B. M, Collin F, Cope L. M, Hobbs B (2003). Summaries of Affymetrix GeneChip probe level data.. Nucleic Acids Res.

[63] Martinat C, Mena I, Brahic M (2002). Theiler's virus infection of primary cultures of bone marrow-derived monocytes/macrophages.. J Virol.

[64] Wagner M, Jonjic S, Koszinowski U. H, Messerle M (1999). Systematic excision of vector sequences from the BAC-cloned herpes virus genome during virus reconstitution.. J Virol.

[65] Ghazal P, Visser A. E, Gustems M, Garcia R, Borst E. M (2005). Elimination of ie1 significantly attenuates murine cytomegalovirus virulence but does not alter replicative capacity in cell culture.. J Virol.

[66] Livak K. J, Schmittgen T. D (2001). Analysis of relative gene expression data using real-time quantitative PCR and the 2(-Delta Delta C(T)) method.. Methods.

[67] Jeon T. I, Zhu B, Larson J. L, Osborne T. F (2008). SREBP-2 regulates gut peptide secretion through intestinal bitter taste receptor signaling in mice.. J Clin Invest.

[68] Fei W, Shui G, Gaeta B, Du X, Kuerschner L (2008). Fld1p, a functional homologue of human seipin, regulates the size of lipid droplets in yeast.. J Cell Biol.

[69] Huang Q, Shen H. M, Shui G, Wenk M. R, Ong C. N (2006). Emodin inhibits tumor cell adhesion through disruption of the membrane lipid Raft-associated integrin signaling pathway.. Cancer Res.

