# Supplementary figures and images for: Host Defense against Viral Infection Involves Interferon Mediated Down-Regulation of Sterol Biosynthesis
Source: PLoS Biol. 2011 Mar 8;9(3):e1000598. doi: 10.1371/journal.pbio.1000598 (PMC3050939; doi:10.1371/journal.pbio.1000598)

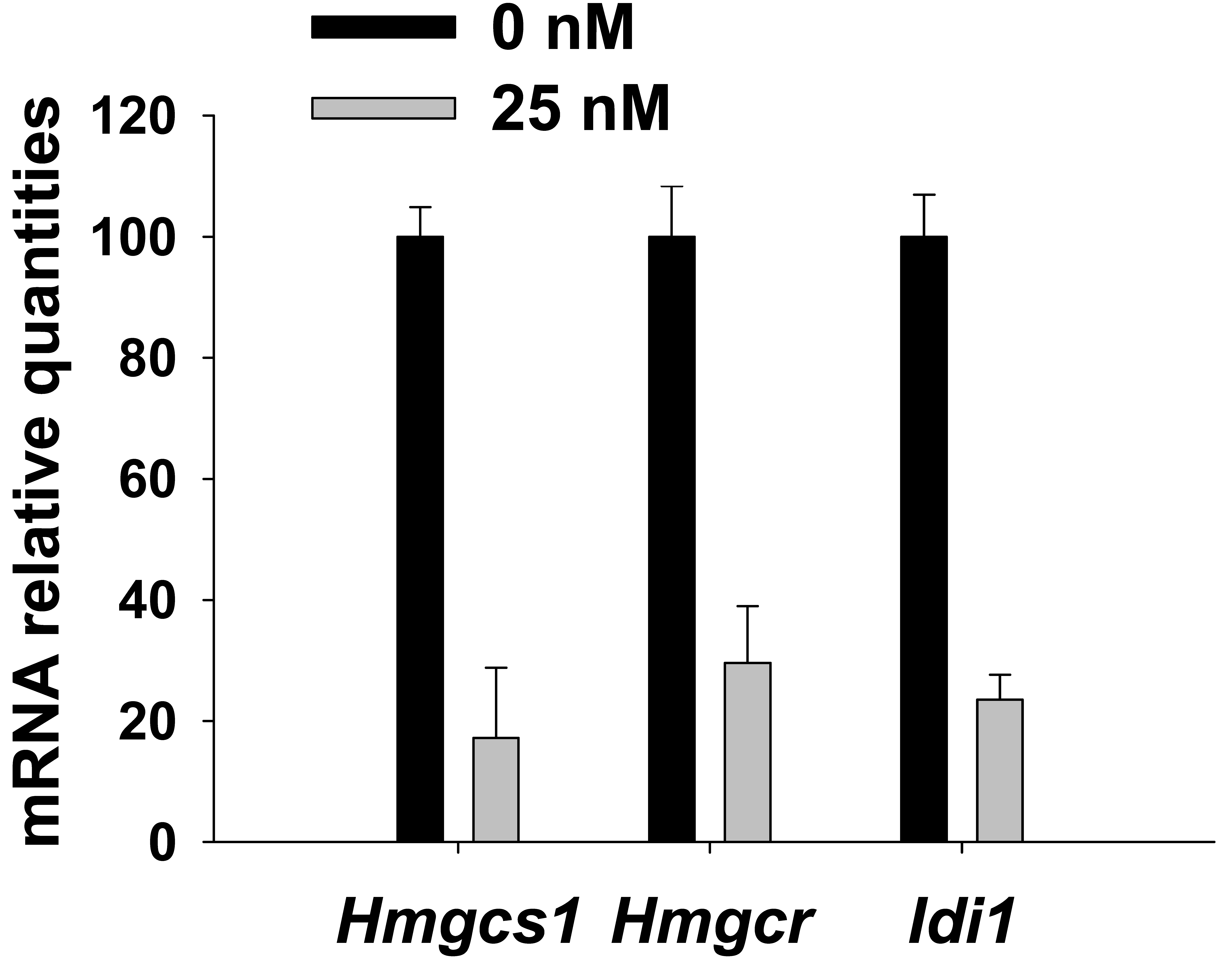

Supplement: Figure S5 — Knock-down efficiency. NIH3T3 cells were transfected with 0.4% Dharmafect 1 and 25 nm of Hmgcs1, Hmgcr, or Idi1 SiRNA smart pool (Dharmacon). After 48 h, RNA was collected and QPCR was performed to check gene expression for Hmgcs1, Hmgcr, and idi1. Gapdh was used for normalization. Hmgcs1 shows an 81% decrease in expression following transfection, Hmgcr a 70% decrease, and Idi1 an 82% decrease. Bars represent means ± SD of triplicate biological measurements. (1.23 MB TIF) [file pbio.1000598.s005.tif]

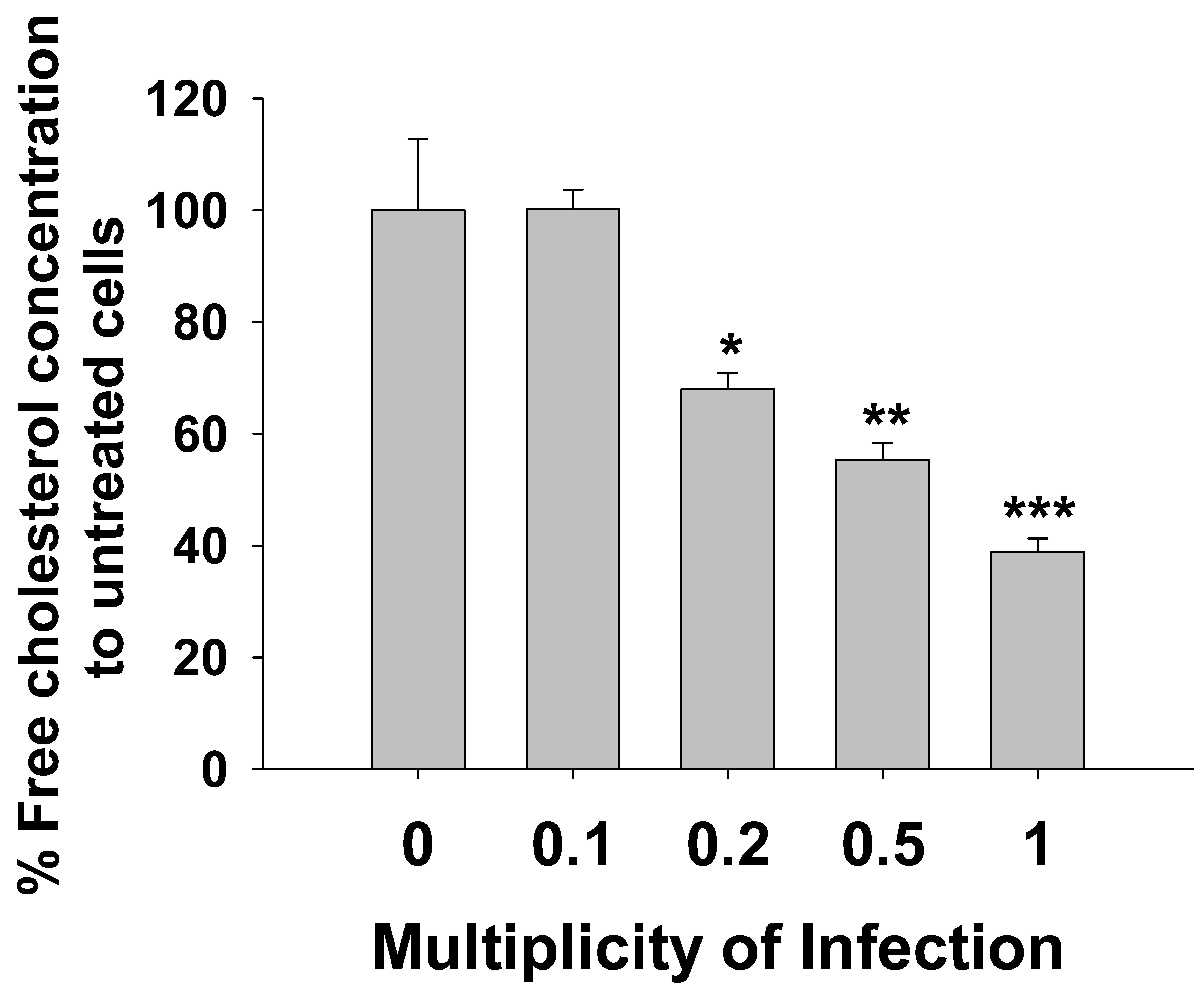

Supplement: Figure S6 — mCMV infection reduced free intra-cellular cholesterol in a dose-dependent manner in BMDM at 48 hpi. BMDM were infected at different MOI (0, 0.1, 0.2, 0.5, and 1) with mCMV virus. Data are represented as the percentage of free intracellular cholesterol concentration from infected cells in comparison to mock treatment. Results represent means ± SD from two independent experiments with triplicate biological measurements for each experiment. (1.32 MB TIF) [file pbio.1000598.s006.tif]

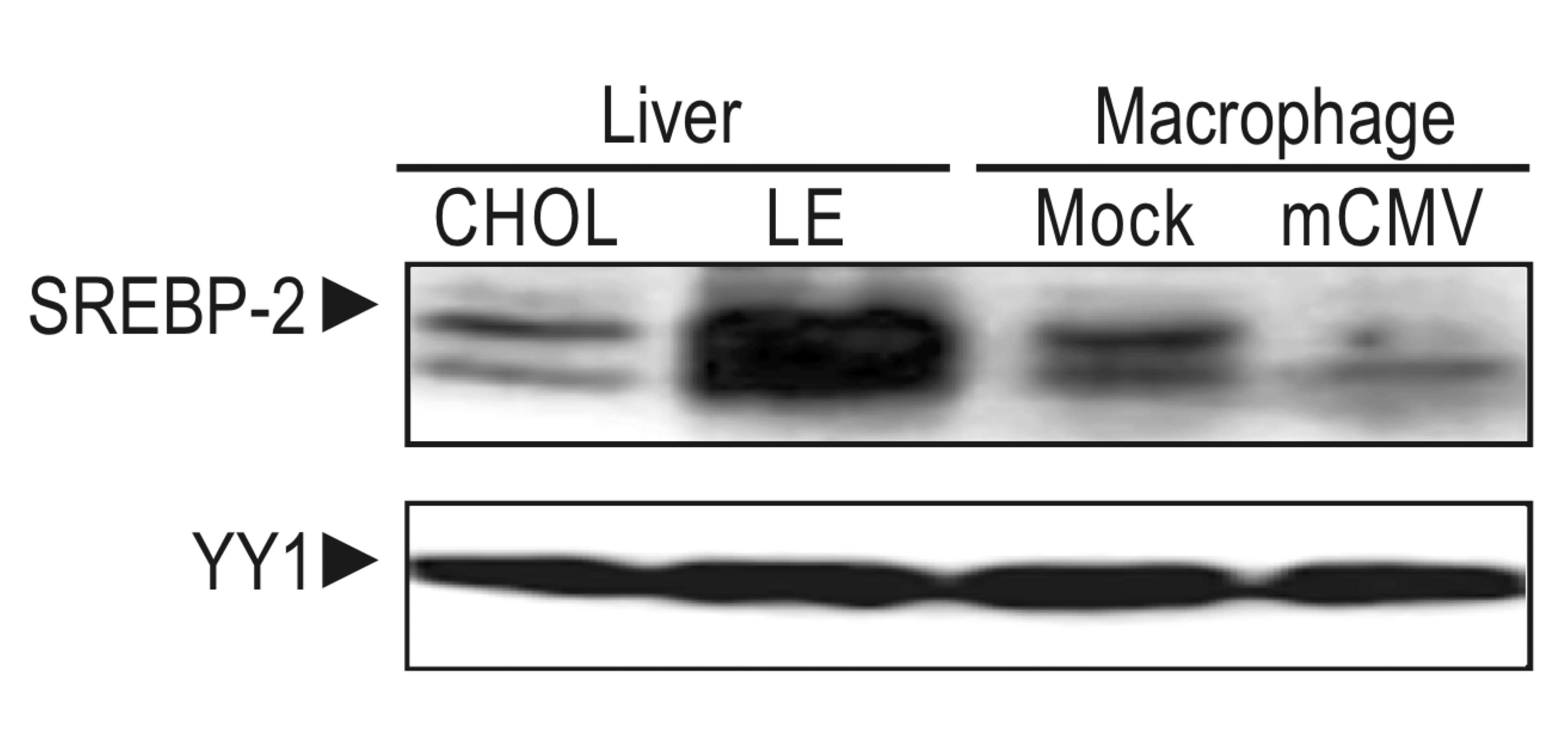

Supplement: Figure S8 — Specificity of the SREBP2 antibody. Lane 1 and 2: Nuclear extract protein prepared from livers of mice fed chow supplemented with either a 2% cholesterol diet (CHOL) or a mixture of lovastatin and ezetimibe were loaded as controls. Arrow indicates the specific SREBP2 cleaved form. As a comparison, lanes 3 and 4 show nuclear protein extracts prepared from macrophages cultured from mock or mCMV infected. YY1 protein was used as a loading control. (1.36 MB TIF) [file pbio.1000598.s008.tif]
