# Supplementary material for: Host Defense against Viral Infection Involves Interferon Mediated Down-Regulation of Sterol Biosynthesis
Source: PLoS Biol. 2011 Mar 8;9(3):e1000598. doi: 10.1371/journal.pbio.1000598 (PMC3050939; doi:10.1371/journal.pbio.1000598)
Supplement: Text S1 — Supporting methods. This file gives an overview of the methods used in this article. (0.13 MB DOC) [file pbio.1000598.s011.doc]

**Supplementary Experimental Details**

**Cell culture and viruses**

All cells and viral stock were routinely mycoplasma tested (Stratagene). NIH/3T3 (ATCC CRL-1658) cells were maintained in Dulbecco's modified Eagle's medium (Invitrogen) supplemented with 10% calf serum and 1% penicillin-streptomycin as previously described[^1]^.

Macrophage cultures were established as described previously[^2]^ from the bone marrow of ten to twelve week old male Balb/c mice (Charles River Laboratories, Kent, U.K.). The animals were maintained in specific pathogen-free conditions at the University of Edinburgh. Housing and animal procedures were approved by the U.K. Government Home Office. Briefly, bone marrow progenitors were flushed from femurs and plated at 8 x 10^5^ cells in 6 well tissue culture dishes (Costar, Corning Inc., NY, USA). The cells were cultured in DMEM/F12 medium supplemented with 10% fetal calf serum (FCS), pen/strep, glutamine and 10% L929 conditioned medium as a source of M-CSF.

**BMDM and NIH/3T3 infection.**

BMDM were infected with the different viruses at a MOI of 1, unless specified in DMEM:F12 3% FCS, 10% L929, and 100 U of penicillin/streptomycin per ml. After 1 hour adsorption, cells were washed in PBS and incubated in fresh DMEM:F12 10% FCS, 10% L929, and 100 U of penicillin/streptomycin per ml.

NIH/3T3 cells were infected with mCMV at a MOI of 1, unless specified, for 1 hour and the cells were washed in PBS and incubated in fresh DMEM 3% FCS, and 100 U of penicillin/streptomycin per ml. SFV (MOI of 10), HSV1 (MOI =1), VV (MOI of 1) and Ad (MOI of 100) were used to infect BMDM for 1 hr in DMEM:F12 3% FCS, 10% L929, and 100 U of penicillin/streptomycin per ml.

**Analysis of lipids using high performance liquid chromatography/mass spectrometry and Electrospray ionization.**

An Agilent high performance liquid chromatography (HPLC) system coupled with an Applied Biosystem Triple Quadrupole/Ion Trap mass spectrometer (4000Qtrap) was used for quantification of individual polar lipids (Phospholipids and sphingolipids). Electrospray ionization-based multiple reaction monitoring (MRM) transitions were set up for the quantitative analysis of various polar lipids[^3]^. HPLC atmosphere chemical ionization (APCI)/MS were carried out for analysis of sterols[^4]^

**RNA extraction from BMDM for QRT-PCR analysis.**

RNA from BMDM cells (Mock, cytokine treated or mCMV infected) in 6-well plates was extracted by washing the cells twice with PBS, adding 200 µl Trizol reagent (Invitrogen, CA) and incubating for 5 min at RT. Samples were then transferred to a 1.5 ml microfuge tube and 40 µl chloroform was added. Samples were incubated at room temperature for 15 min before being centrifuged (13,000 rpm, 4ºC, 5 min), aqueous phase was collected, and an equal volume of chloroform was added and samples were centrifuged (13,000 rpm, 4ºC, 5 min). The upper aqueous layer was removed and 0.1 volumes 3 M NaOAc, 2.5 volumes EtOH were added. Samples were incubated (-80ºC, 1 hour) and then centrifuged (13,000 rpm, 4ºC, 30 min). Supernatant was removed and the pellets washed in 200 µl 70% (v/v) ethanol and centrifuged as before. This step was repeated twice. RNA pellets were then resuspended in 50 µl RNAse-free H_2_O. RNA quantity and integrity was assayed by respectively using a Nanodrop ND-1000 (Nanodrop Technologies, DE) to measure the A260/A230 and the A260/A280 ratio (only samples with a ratio A260/230 ≥ 1.8 and a A260/280 ≥ 2 were selected) and the 2100 Bioanalyzer (Agilent); all RNA samples used for analysis had an RNA integrity number (RIN number) of >9.0, indicating high-quality RNA with minimal degradation products.

**SREBP2 immunoblot.**

Nuclear protein extracts were prepared from macrophages cultured as described above and 25 µg from mock, CMV infected or IFN treated samples were loaded on 8% SDS-PAGE gels run at a constant 100V in 1x Tris-Glycine/0.25% SDS running buffer. Nuclear extract protein (5 ug) prepared from livers of mice fed chow supplemented with either a 2% cholesterol diet (CHOL) or a mixture of lovastatin and ezetimibe as described described by Jeon et al 2008 were loaded as controls. The protein from the gels were transferred onto 0.22 µm nitrocellulose membranes using 1x Tris-Glycine/20% methanol transfer buffer run at 350 mA for 1h. Membranes were stained with Ponceau S solution (Sigma) to confirm transfer and blocked for 1h at room temperature in a 5% non-fat dry milk/TBS solution. Membranes were probed overnight at 4°C in a 2% non-fat dry milk/TBS/0.05% Tween-20 solution containing 2 µg/ml rabbit-anti-mouse SREBP-2 polyclonal antibody. The anti-mouse SREBP-2 polyclonal antibody is a custom antibody raised against a recombinant fragment of the mature mouse SREBP-2 was described previously[5]. Following overnight incubation in the primary antibody, membranes were washed 3 x 10 min in TBS/0.05% Tween-20 at room temperature. Membranes were probed with appropriate goat-anti-species-HRP secondary antibody for SREBP-2 and beta-actin loading control for 1h at room temperature, followed by 3 x 10 min washes in TBS/0.05% Tween-20. Immunoblots were visualized using x-ray film exposure and the SuperSignal West Femto (SREBP-2) or Pico (beta-actin) chemiluminescent reagent.

**Characterization of BMDM by flow cytometry.**

Maturation of BMDM cells was tested by flow cytometry analysis for the expression of murine proteins specific for mature macrophages. Analysis was undertaken for F4/80 (Caltag Laboratories, UK) and CD11b (eBiosciences, UK). Analyses were performed using a FACScan. More than 93% of the cells were positive for both markers and therefore considered as mature macrophages (data not shown).

**Cell viability**

For cytokine, Simavstatin, mevalonate and Gancyclovir treatments and SiRNA transfection, cell viability was assessed using the Cell Titer Blue (CTB) assay (Promega, UK) at different time point of the treatments.

**Micro array experiment and analysis**

***Temporal gene expression analysis of BMDM***

*Experiment*

Briefly, BMDM were either mock treated or infected with mCMV at a multiplicity of infection (MOI) of 1 or treated with 10 U IFNγ (Boelinger Manhaim Corp). Every 30 min for each treatment, cells were lysed with Trizol and stored at -80ºC (a full description of the experiment will be discussed elsewhere).

RNA was extracted from each sample using Trizol RNA extraction protocol. Mock samples were pooled and labelled with Cy3 while the lyzed or IFNγ treated samples were labelled with Cy5 using a modification of the Agilent Fluorescent protocol, using half of the standard Cy3/Cy5 labelled dUTP concentration. The Cy3 labelled pooled control was hybridized with each of the 75 Cy5 labelled samples according to the Agilent Low RNA Input protocol. The dual hybridizations were carried out on Mouse Agilent V2 array (G4121A, 20868 annotated probes), and were scanned on an Agilent Technologies scanner. Agilent feature extraction software (V.A7.5.1) was used to extract numeric data for further analysis.

*Processing microarray data*

Data of each sample was first background corrected, and then transformed to log (base 2) scale. Any non-positive values in the background corrected data were replaced with unity before performing the log-transformation. Exploratory plots do not suggest any intensity dependent non-linear trend in the data. Therefore subset median normalization based on 42 positive controls needed to be used to remove any chip-to-chip variation within the data. More specifically, suppose *(m_1_, m_2,..._, m_50_)* represent the medians of the positive controls for the 50 arrays, and m0 is their mean. Then the normalizing constants for the 50 samples are defined as *(m_0_-m_1_, m_0_-m_2 ..._, m_0_-m_50_)*. The data of each sample are adjusted by adding these constants to the log (base 2) intensity data of the respective samples.

The normalised data was filtered using ROC analysis based on 42 positive and 111 negative controls. For each array, a threshold value was calculated corresponding to the 80% sensitivity level of the corresponding ROC curve. A gene is declared on in a particular sample (time point) if its log2 expression value exceeds that threshold. Genes are then selected for further analysis if they were found to be on in 5 or more consecutive time points in a particular experimental condition.

*Statistical analysis of time course*

The method below provides statistical test for identifying within condition temporal differential expression as well as test for identifying differential expression between conditions. The framework of the model is defined as

*y_ij_ = µ_i_ (t_j_) + ε_ij_* (1)

with *y_ij_* being the log (base 2) expression level of gene *i* in sample *j* and the sample *j* is observed at time point *t_j_* , where there are *i* = 1,2,...,M genes on each array and *J* =1,2,...,N time points/samples for each experimental conditions. The population average time curve is represented by a linear regression of a p-dimensional basis:

*µ_i_ (t)* = α_i_ + β_i_^T^ S(t) = α_i_ + β_i1_S_1_ (t) + β_i2_S_2_ (t) + ... + β_ip_S_p_(t) (2)

where α_i_ is the gene-specific intercept term, β_i_ = β_i1_, β_i2_,..., β_ip_)^T^ is a p-dimensional vector of regression parameters, and S(t) = S_1_ (t), S_2_(t), ..., S_p_(t))^T^ is a known p-dimensional basis.

A polynomial of degree p or more flexible natural cubic splines has been suggested as a choice of basis. For identifying temporal differential expression, the null hypothesis, restricting *µ_i_ (t)* to be constant, can be tested against the alternative hypothesis that *µ_i_ (t)* is a curve. Thus, mathematically, the hypothesis of no temporal differential expression for gene *i* can be stated as

H_0_: β_i_ = 0, or equivalently, H_0_: *µ_i_* = α_i_ (3).

The observed statistics and null statistics were used to estimate a q-value for each gene, which estimate the false discovery rate for calling the gene significant.

In order to select very highly significant temporal changes, we considered a q-value cut off of 10-^6^.

***Cholesterol gene expression profiling of SFV HSV1 VV and AD infected macrophages***

*Experiment*

The avirulent Semliki Forest virus strain A774 (SFV A7), HSV1 (strain), Vaccinia virus and Adenovirus 2 were used to infect BMDM.

RNA was isolated from cultured macrophages using the TRIzol method according to the manufacturer’s instructions (Invitrogen, San Diego, CA, USA). RNA concentration and purity were obtained by spectrophotometry and RNA integrity monitored using the Agilent 2100 bioanalyzer system (Agilent, Palo Alto, CA, USA). Five micrograms of total RNA isolated from macrophage cultures were used to synthesise biotinylated cRNA target which was hybridised to the Mouse Genome 430 2.0 GeneChip (Affymetrix, Santa Clara, CA, USA). RNA was reverse transcribed using Superscript II reverse transcriptase (Invitrogen) into double stranded cDNA using oligo dT primers that contained a T7 promoter. cDNA was extracted with phenol-chloroform and a Phase Lock Gel (Eppendorf, Hamburg, Germany) and precipitated with ethanol and ammonium acetate. Biotinylated cRNA was synthesised using the cDNA as a template in an in vitro transcription reaction using the BioArray HighYield RNA Transcript Labelling Kit (Enzo Life Sciences Inc., Farmingdale, NY, USA) as described by the manufacturer. The resulting biotinylated target cRNA was purified using RNeasy columns according to the manufacturers instructions (QIAgen Ltd., Crawley, UK) and quantified by spectrophotometry. 15µg of purified biotinylated cRNA was fragmented by heating for 35 mins at 94^o^C in the presence of magnesium ions, spiked with eukaryotic hybridisation control and hybridised to Mouse Genome 430 2.0 microarrays overnight at 45^o^C. After hybridisation the array was washed, stained with phycoerythrin coupled streptavidin and processed on the Affymetrix GeneChip Fluidics Workstation 400 using the EukGE-Ws2v4 protocol. Microarrays were then scanned using the Agilent 2500A GeneArray Scanner (Agilent).

*Data processing and statistical analysis*

Data from hybridised chips were acquired using proprietary Affymetrix platform scanners and GCOS software (Affymetrix). The numeric data were processed and subsequently analysed with the Bioconductor package for the R statistical programming environment[6]. Raw data distributions and summary statistics were assessed for quality. Data were then background corrected, quantile normalised and probe-set summarised using the RMA algorithm[7]. Prior to statistical analysis, a non-specific filter was applied to remove genes that were not expressed on any of the samples in the experiment. Null hypotheses for each gene were based on the comparison between mock arrays and each of the 3 biological conditions; they were tested using an empirical Bayes test, providing good robustness for small sample sizes[8]. In order to adjust for multiple testing issues, the false discovery rate was controlled using the Benjamini-Hochberg p-value adjustment method. Genes were interpreted on the basis of differential expression between mock and each of the 3 groups, and the corresponding statistical significance. Visualisation of data was performed using GeneSpring GX v7.3 (Agilent).

***Nascent RNA expression profiling of TYK2 -/- mCMV infected macrophages***

*Experiment*

Bone marrow-derived macrophages were isolated and grown in the presence of Csf1 derived from L929 cells as described[2] except cells were cultivated in 15cm diameter tissue culture plates for 7 days before treatment.

Incorporation of 4-thiouridine (Sigma) into nascent RNA was undertaken as described by Dölken *et al*[9]*.* In brief, at 360 minutes post-infection, 10mls medium was aspirated from all plates, added to 80µl 4-Thiouridine, mixed and immediately returned to the culture dish. After 30 minutes, to end the RNA labelling period, terminate transcription and lyse the cells, medium was aspirated from the labelled BMDM and replaced with 4mls of RLT lysis buffer (Qiagen).

Total RNA was isolated using an RNeasy Midi kit (Qiagen) according to manufacturers instructions, quantitated using a Nanodrop (Thermo Scientific) and integrity was confirmed using an Agilent Bioanalyser (Agilent UK).

Newly transcribed RNA (ntRNA) was then isolated as described in Dölken *et al.*[9] and again quantitated using a Nanodrop.

*Newly transcribed RNA labelling and Microarray Analysis*

Processing of ntRNA samples (94ng) for hybridisation to Affymetrix Mouse Gene 1.0 ST arrays was undertaken according to manufacturers instructions (Affymetrix). Hybridisation, washing, staining and scanning of the arrays were also undertaken following standard Affymetrix protocols.

After scanning and data capture, Partek (Partek Incorporated, USA) was used to implement all quality control and statistical analyses. In brief, Affymetrix control criteria were considered and found to be consistent with manufacturers recommendations. For the purposes of presentation, gene expression values for the specific genes of interest from control (mock infected) BMDM were adjusted to a value of 1. Values for expression in infected cells (white) were then expressed as a number relative to the control.

**Bioinformatics *analysis***

***Generation of lipid associated genes list.***

In order to create a database of lipid associated genes list, we used a published database of 1224 proteins associated to LIPID MAPS proteome database (http://www.lipidmaps.org)[10]. LMPD is an object-relational publicly available database of lipid-associated protein sequences and annotations using UniProt, EntrezGene, ENZYME, GO, KEGG and other public resources. Using web resources: Netaffx (http://www.affymetrix.com), DAVID (http://david.abcc.ncifcrf.gov)[11] and NCBI website, we could created a list of 1080 LAGs associated with LMPD id, gene symbol, uniprot Id, unigen ID, entrez ID, David ID, Affy ID, Agilent ID, lipids categories and gene full name is provided in **supplementary file 1**.

***Selection of significantly regulated LAGs***

In order to filter the LAGs significantly regulated in response to mCMV infection and IFNγ treatment, we applied a 10^-6^ q-value threshold (see temporal differential analysis of time course in supplementary materials and methods). From the 958 (89%) LAGS expressed in our microarray dataset. 195 LAGs (20% of the 958 genes) were considered as significantly regulated by mCMV infection and 113 LAGs (12% of the 958 genes) were considered as significantly regulated by IFNγ treatment.

***Clustering analysis of selected regulated LAGs.***

Normalized expression data was clustered using Pearson correlation (r>0.9) to classify

genes as up or down regulated by either treatment. For mCMV treatment, 62 LAGs (6.5% of the 958 original LAGs) were clustered as down regulated (**Tab. S1A**) and 133 LAGs (14% of the 958) were clustered as up regulated (**Tab. S1B**). For IFNγ treatment, 51 LAGs clustered as down regulated (**Tab. S1C**) (5% of the 958) and 65 LAGs were clustered as up regulated (7% of 958) (**Tab. S1D**). Venn diagram analysis (data not shown) was used to identify LAGs regulated by mCMV infection and INFγ treatment. 35 LAGS (**Tab. S1E**) were identified as being down regulated by mCMV infection and IFNγ treatment and 47 LAGs (**Tab. S1F**) were identified as being up regulated by mCMV infection and INFγ treatment.

***Lipid class analysis***

In order to study the representation of lipids class associated with the regulated LAGs by mCMV infection or INFγ treatment or both, genes were grouped by lipid class according to LMPD classification (http://www.lipidmaps.org)[10] and results are represented as pie charts **Figure S1A-C**.

***Canonical pathway analysis.***

Significantly altered LAGs lists were used to perform a canonical pathways analysis using Ingenuity© Pathway Analysis (IPA, http://www.ingenuity.com) IPA canonical pathways analysis identified the most significant known biological pathways for a given set of genes using the Ingenuity Pathways Knowledge Base (IPKB). The significance of a canonical pathway is controlled by P value, which is calculated using the right-tailed Fisher’s exact test. The significance threshold of a canonical pathway is set to 1.3, which is derived by -log10 [P value], with P≤0.05. Ratios represent the number of LAGs represented in the total number of genes present in the given canonical pathway. DAVID was also used as an independent assessment of pathway analysis.

Metabolite abbreviations in Fig 1A.

ACoA: acetyl – CoA

AaCoA: Acetoacetyl-CoA

HCoA: HMG-CoA

M: mevalonate

M5P: mevalonate-5P

M5PP: mevalonate-5PP

IsPP: isopentyl-PP

FPP: farnesyl-PP

Squa: squalene

23Ox: 2,3 oxydosqualene

Lan: lanesterol

44Di: 4,4 dimethyl-cholesta-8,14,24-trienol

14De: 14-demethyl-lanosterol

4MZC: 4-methylzymosterol-carboxylate

3K4M: 3-keto-4-methyl-zymosterol

4MZ: 4-methylzymosterol

Zym: zymosterol

Cho8: cholesta-8,en-3beta-ol

Lath: lathosterol

7DeC: 7-dehydro-cholesterol

Cho7: cholesta-7,24-dien-3beta-ol

7DeD: 7-dehydro-desmosterol

Des: desmosterol

Chol: cholesterol

**References**

1. Ghazal P, Visser AE, Gustems M, Garcia R, Borst EM, et al. (2005) Elimination of ie1 significantly attenuates murine cytomegalovirus virulence but does not alter replicative capacity in cell culture. J Virol 79: 7182-7194.

2. Martinat C, Mena I, Brahic M (2002) Theiler's virus infection of primary cultures of bone marrow-derived monocytes/macrophages. J Virol 76: 12823-12833.

3. Fei W, Shui G, Gaeta B, Du X, Kuerschner L, et al. (2008) Fld1p, a functional homologue of human seipin, regulates the size of lipid droplets in yeast. J Cell Biol 180: 473-482.

4. Huang Q, Shen HM, Shui G, Wenk MR, Ong CN (2006) Emodin inhibits tumor cell adhesion through disruption of the membrane lipid Raft-associated integrin signaling pathway. Cancer Res 66: 5807-5815.

5. Jeon TI, Zhu B, Larson JL, Osborne TF (2008) SREBP-2 regulates gut peptide secretion through intestinal bitter taste receptor signaling in mice. J Clin Invest 118: 3693-3700.

6. Team RDC (2010) R: A Language and Environment for Statistical

Computing.

7. Irizarry RA, Hobbs B, Collin F, Beazer-Barclay YD, Antonellis KJ, et al. (2003) Exploration, normalization, and summaries of high density oligonucleotide array probe level data. Biostatistics 4: 249-264.

8. Smyth GK (2004) Linear models and empirical bayes methods for assessing differential expression in microarray experiments. Stat Appl Genet Mol Biol 3: Article3.

9. Dolken L, Ruzsics Z, Radle B, Friedel CC, Zimmer R, et al. (2008) High-resolution gene expression profiling for simultaneous kinetic parameter analysis of RNA synthesis and decay. RNA 14: 1959-1972.

10. Cotter D, Maer A, Guda C, Saunders B, Subramaniam S (2006) LMPD: LIPID MAPS proteome database. Nucleic Acids Res 34: D507-510.

11. Huang da W, Sherman BT, Tan Q, Kir J, Liu D, et al. (2007) DAVID Bioinformatics Resources: expanded annotation database and novel algorithms to better extract biology from large gene lists. Nucleic Acids Res 35: W169-175.
